# Supplementary material for: Visual prognosis and complications of congenital ectopia lentis: study protocol for a hospital-based cohort in Zhongshan Ophthalmic Center
Source: BMJ Open. 2023 Jun 27;13(6):e072542. doi: 10.1136/bmjopen-2023-072542 (PMC10351230; doi:10.1136/bmjopen-2023-072542)
Supplement: Supplementary data [file bmjopen-2023-072542supp001.pdf]

Supplemental table 1. Complete overview of follow-up and examinations.

Examination	Time points of non-surgical group				Time points of surgical group				
	Baseline	1 y	2 y	3 y	Preoperation*	3 m	1 y	2 y	3 y
Demographic data	✓				✓				
Slit-lamp examination	✓	✓	✓	✓	✓	✓	✓	✓	✓
Visual acuity and refraction	✓	✓	✓	✓	✓	✓	✓	✓	✓
Axial length	✓	✓	✓	✓	✓	✓	✓	✓	✓
High-order aberrations	✓	✓	✓	✓	✓	✓	✓	✓	✓
Specular microscopy					✓	✓	✓	✓	✓
Anterior segment OCT	✓	✓	✓	✓	✓	✓	✓	✓	✓
UBM	✓	✓	✓	✓	✓				
Slit-lamp photography	✓	✓	✓	✓	✓				
Pentacam	✓	✓	✓	✓	✓	✓	✓	✓	✓
IOP	✓	✓	✓	✓	✓	✓	✓	✓	✓
Echocardiography	✓	✓	✓	✓	✓		✓	✓	✓
BMI	✓	✓	✓	✓	✓		✓	✓	✓
Hand radiograph	✓	✓	✓	✓	✓		✓	✓	✓
Posterior segment OCT	✓			✓	✓				✓
Gene detection†	✓				✓				
PedEyeQ	✓			✓	✓				✓
Adverse events	✓	✓	✓	✓	✓	✓	✓	✓	✓

** Preoperation examninations will not be repeated if the patient has just completed follow-up and meets the surgical indications.*

† Gene detection will not be repeated if the patient already has a valid genetic testing report.

OCT, optical coherence tomography; UBM, ultrasound biomicroscopy; IOP, intraocular pressure; BMI, body mass index; PedEyeQ, the Pediatric Eye Questionnaire; y, years; m, months.
