# Supplementary material for: Visual prognosis and complications of congenital ectopia lentis: study protocol for a hospital-based cohort in Zhongshan Ophthalmic Center
Source: BMJ Open. 2023 Jun 27;13(6):e072542. doi: 10.1136/bmjopen-2023-072542 (PMC10351230; doi:10.1136/bmjopen-2023-072542)
Supplement: Author's manuscript [file bmjopen-2023-072542.draft_revisions.pdf]

BMJ Open

BMJ Open is committed to open peer review. As part of this commitment we make the peer review history of every article we publish publicly available.

When an article is published we post the peer reviewers' comments and the authors' responses online. We also post the versions of the paper that were used during peer review. These are the versions that the peer review comments apply to.

The versions of the paper that follow are the versions that were submitted during the peer review process. They are not the versions of record or the final published versions. They should not be cited or distributed as the published version of this manuscript.

BMJ Open is an open access journal and the full, final, typeset and author-corrected version of record of the manuscript is available on our site with no access controls, subscription charges or pay-per-view fees (http://bmjopen.bmj.com).

If you have any questions on BMJ Open's open peer review process please email info.bmjopen@bmj.com

BMJ Open

Visual Prognosis and Complications of Congenital Ectopia Lentis: A Prospective Follow-up Cohort Study

Journal:	*BMJ Open*
Manuscript ID	bmjopen-2023-072542
Article Type:	Protocol
Date Submitted by the Author:	05-Feb-2023
Complete List of Authors:	Xu, Pusheng; Sun Yat-Sen University Zhongshan Ophthalmic Center State Key Laboratory of Ophthalmology<br>Ng, Kityee; Sun Yat-Sen University Zhongshan Ophthalmic Center State Key Laboratory of Ophthalmology<br>Jin, Ling; Sun Yat-Sen University Zhongshan Ophthalmic Center State Key Laboratory of Ophthalmology<br>Young, Charlotte; Albany Medical College<br>Liu, Siyuan; Sun Yat-Sen University Zhongshan Ophthalmic Center State Key Laboratory of Ophthalmology<br>Liang, Xiaolin; Sun Yat-Sen University Zhongshan Ophthalmic Center State Key Laboratory of Ophthalmology<br>Liu, Zhenzhen; Sun Yat-Sen University Zhongshan Ophthalmic Center State Key Laboratory of Ophthalmology<br>Zhang, Xinyu; Sun Yat-Sen University Zhongshan Ophthalmic Center State Key Laboratory of Ophthalmology<br>Jin, Guangming; Sun Yat-Sen University Zhongshan Ophthalmic Center State Key Laboratory of Ophthalmology<br>Zheng, Danying; Sun Yat-Sen University Zhongshan Ophthalmic Center State Key Laboratory of Ophthalmology,
Keywords:	Cataract and refractive surgery < OPHTHALMOLOGY, Medical ophthalmology < OPHTHALMOLOGY, Paediatric ophthalmology < PAEDIATRIC SURGERY, Ophthalmology < SURGERY

SCHOLARONE™
Manuscripts

Visual Prognosis and Complications of Congenital Ectopia Lentis: A Prospective Follow-up Cohort Study

Authors

Pusheng Xu, MD[1†]

Kityee Ng, MD[1†]

Ling Jin, MSc[1]

Charlotte Aimee Young, BS[2]

Siyuan Liu, MD[1]

Xiaolin Liang, MD[1]

Zhenzhen Liu, MD, PhD[1]

Xinyu Zhang, MD, PhD[1]

Guangming Jin, MD, PhD[1*]

Danying Zheng, MD[1*]

Institutions

1. State Key Laboratory of Ophthalmology, Zhongshan Ophthalmic Center, Sun Yat-sen University, Guangdong Provincial Key Laboratory of Ophthalmology and Visual Science, Guangdong Provincial Clinical Research

For peer review only

Center for Ocular Diseases, Guangzhou 510060, China.

2. Albany Medical College, 49 New Scotland Ave Albany, New York, Unite States.

† Pusheng Xu and Kityee Ng are joint first authors.

***Corresponding Author:**

Guangming Jin, MD, PhD.

State Key Laboratory of Ophthalmology, Zhongshan Ophthalmic Center, Sun Yat-sen University, Guangdong Provincial Key Laboratory of Ophthalmology and Visual Science, Guangdong Provincial Clinical Research Center for Ocular Diseases, Guangzhou 510060, China.

Email: jingm@mail2.sysu.edu.cn

Professor Danying Zheng, MD.

State Key Laboratory of Ophthalmology, Zhongshan Ophthalmic Center, Sun Yat-sen University, Guangdong Provincial Key Laboratory of Ophthalmology and Visual Science, Guangdong Provincial Clinical Research Center for Ocular Diseases, Guangzhou 510060, China.

Email: zhengdyy@163.com

Financial support

This study was supported by the National Natural Science Foundation of China (81873673, 81900841), and the Basic and Applied Basic Research

For peer review only

Foundation of Guangdong Province (2021A1515011673, 2022A1515011181).

Disclosures: None of the authors has a financial or proprietary interest in any material or method mentioned.

Number of Figures: 2

Word count: 2803

For peer review only

Visual Prognosis and Complications of Congenital Ectopia Lentis: A Prospective Follow-up Cohort Study

ABSTRACT

Introduction Congenital ectopia lentis (CEL) is a rare ocular disease characterized by the dislocation or displacement of the lens. Patients with a mild degree of lens dislocations can be treated with conservative methods (e.g., corrective eyeglasses or contact lenses). In contrast, patients with severe CEL are usually treated with surgical treatment. However, few studies have focused on the visual prognosis and complications in conservative and surgical treatment patients. This study aims to investigate the prognosis and complications in CEL patients with conservative and surgical treatment, which is vital for CEL management, especially the choice of surgical timing and surgical method.

Methods and analysis A follow-up cohort study will be conducted at Zhongshan Ophthalmic Center. All patients diagnosed with congenital ectopia lentis and aged ≥ 3 years old will be enrolled. Patients with mild lens subluxation and stable visual conditions will be included in the non-surgical group and follow-up at 1, 2 and 3 years after enrollment. Patients with severe lens subluxation that accept CEL surgery will be included in the surgical group. Different surgical techniques will be used depending on the severity of dislocation. Patients will be followed up at 3, 6 months, and 1, 2 and 3 years postoperatively. Over a 5-year follow-up period, patients will receive a detailed ocular examination, including optometry, biological measurement, endokeratoscope, ultrasound biomicroscopy, anterior segment and posterior segment optical coherence tomography (OCT), optical coherence tomography angiography (OCTA), echocardiography and questionnaires on vision-related quality of life. The primary outcome is the change of best corrected visual acuity and the incidence of complications in both groups.

Ethics and dissemination Ethics approval was obtained from the ethics

committee of the Zhongshan Ophthalmic Center (number: 2022KYPJ207). Study findings will be published in a peer-reviewed journal.

Trial registration number NCT05654025.

For peer review only

Strengths and limitations of this study

- This study is the first follow-up cohort of congenital ectopia lentis (CEL) in the Chinese population.
- Long-term follow-up will provide valuable evidence for CEL management, especially the choice of surgical timing and surgical method.
- Detailed examinations will help to more accurately understand the present landscape of patients with CEL and provide a basis for personalized examinations and treatments in the future.

For peer review only

INTRODUCTION

Congenital ectopia lentis (CEL) is a rare ocular disease characterized by the dislocation or displacement of the lens. The prevalence rate of CEL is about 6.4/100,000.[1] Several systemic diseases have been reported to be associated with CEL, such as Marfan syndrome, homocystinuria, Weill-Marchesani syndrome and Ehlers-Danlos syndrome.[2]

Patients with mild lens dislocations can be treated with conservative methods (e.g., corrective eyeglasses or contact lenses).[3] However, progressive subluxation or complete dislocation of the lens can cause a high degree of myopia or astigmatism, even amblyopia.[4] Surgical intervention is crucial in the management of patients with severe CEL. Currently, there is no unified standard for operation timing for CEL. A previous study recommended surgical intervention when the near vision of children was less than 0.4.[5] However, some researchers suggested that surgery should be performed when the best corrected visual acuity (BCVA) is less than 0.3 or monocular diplopia occurs.[4 6] Few studies have focused on the refractive change and visual prognosis in CEL patients both with conservative treatment and surgical treatment.[7]

Several surgical techniques have been reported over the past decades, such as lensectomy, phacoemulsification without intraocular lens (IOL) implantation, phacoemulsification and IOL implantation (with or without capsular tension ring) and various transscleral fixation of IOL.[8-10] However, the safety and efficacy of these techniques have not been fully validated so far, especially in the Chinese population. Herein we will conduct this prospective follow-up cohort study at the Zhongshan Ophthalmic Center, one of the biggest ophthalmic hospitals in China.[11] All children diagnosed with CEL will be followed up for at least three years. Long-term changes in best-corrected visual acuity and the incidence of complications will be evaluated in patients with conservative treatment and surgical treatment.

METHODS AND ANALYSIS

For peer review only

This study will be conducted from 5 December 2022 to 5 December 2027. The study was registered on Clinicaltrials.gov (NCT05654025).

Objective

The study is designed as a prospective clinical trial to investigate the prognosis and complications in CEL patients with conservative and surgical treatment. The aim is to serve as a reference for disease management, specifically for choice of surgical timing and surgical method.

Study design

An approximately 5-year longitudinal cohort study will be conducted at Zhongshan Ophthalmic Centre, Guangzhou, China. This study will adhere to the Declaration of Helsinki, and ethics have been approved by the ethics committee of the Zhongshan Ophthalmic Center (number: 2022KYPJ207). This study is designed used the SPIRIT reporting guidelines.[12]

Eligibility criteria

Inclusion criteria

1. Diagnosed with congenital lens dislocation and age ≥ 3 years old.

2. Agree to participate in this study with written informed consent from patients or legal guardians.

Exclusion Criteria

1. History of ophthalmic trauma or other ophthalmic surgeries.

2. Combined with other ophthalmic diseases such as glaucoma, uveitis and corneal disease.

3. Patients who could not cooperate in the examinations.

For peer review only

Study setting and participants

This clinical trial will be conducted at Zhongshan Ophthalmic Center, one of the largest ophthalmic hospitals in China. This study aims to investigate the visual prognosis and complications in CEL patients with different treatments. Patients aged three years or above diagnosed with CEL will be recruited from Zhongshan Ophthalmic Center. Moreover, each participant will be followed for at least three years.

Once the participants meet the requirement of our eligibility criteria, they will be asked to join the WeChat (an instant messaging tool) group on the phone and be provided with informed consent. Interested participants or their guardians will sign the consent form and, if appropriate, will complete a thorough ocular examination and systemic evaluations.

Recruitment

The outpatient clinics will carry out the first screening at Zhongshan Ophthalmic Center. Potential participants will be further confirmed by eligibility and recruited at Zhongshan Ophthalmic Center for clinical trials. One of our researchers will contact the participants and explain the trial process in detail to ensure the participants or guardians fully understand the whole study. Once they agree and sign the consent form, further information will be provided, including the purpose of the study, examinations, the importance of follow-up time and duration, and possible risk in treatment. Then the trial will proceed subsequently.

Sample size

Sample size will be estimated as follows: Assuming the incidence of complications in CEL patients is 15%, and the margin of error is 20%. For a 5% significance level, $Z_{\alpha/2}$ is 1.96 for the two-tailed alternative hypothesis. Sample size = $(Z_{\alpha/2})^2 \cdot P(1-P)*1/E^2 = (1.96)^2 \cdot 15\% \cdot (1-15\%)/(15\% \cdot 20\%)=544$. Assuming

For peer review only

the loss ratio of 10%, the adjusted sample size will be 544/(1-0.1) = 544/0.9= 604.

Preoperative management

All patients diagnosed with CEL will be followed up since the enrollment. The surgery will be performed when either of the following situations occurs: 1) BCVA < 0.3; 2) monocular diplopia; 3) progressive subluxation of the lens affecting the pupillary axis; 4) complicated with severe cataract or secondary glaucoma or corneal endothelial decompensation or retinal detachment.[7]

Preoperative medication

All patients will be routinely administered levofloxacin eye drops (four times a day for three days) before surgery to minimize the risk of infection. An intramuscular injection of ethamsylate (1mg/kg)[13] will be used 30 minutes before surgery to reduce bleeding.

Anaesthesia

General anaesthesia or retrobulbar anaesthesia will be used according to the standard clinical routine.

Surgery methods management

The choice of surgical methods depends on the degree of ectopia lentis and the state of zonules. If the extent of the unhealthy zonules (broken or weak) ≤ 180°, phacoemulsification and in-the-bag IOL implantation (with or without capsular tension ring) will be used. Otherwise, the capsular bag will be removed, and IOL will be fixed through transscleral fixation. Surgery will be performed by the same surgeon (Dr. DY Zheng). Rayner 920H/970C or Sensar AR40e will be used as the implanted IOL.

The surgical techniques for patients who received in-the-bag IOL implantation are as follows: A 3.0 mm corneal tunnel incision will be made at

For peer review only

12 o'clock. Then, a continuous circular capsulorhexis will be performed manually. Iris retractors will stabilize the bag, and the lens will be aspirated with a phacoemulsifer. IOL will be implanted in-the-bag, and the capsular tension ring will be implanted when the IOL cannot be stably fixed.

For patients who received transscleral fixation of IOL, the surgical techniques will be performed as the previous study described.[7] In brief, a 3.0 mm corneal tunnel incision will be made at 12 o'clock. Then, a continuous circular capsulorhexis will be performed manually, and the capsular bag will be removed after the phacoaspiration. Transscleral fixation of IOL will be performed with the two IOL haptic sutured by 8-0 polypropylene at 2.0 mm posterior to the corneal limbus. Anterior vitrectomy will be performed when severe vitreous prolapse occurs.

Postoperative management

After surgery, topical anti-inflammatory and anti-infective treatment will be routinely administered. If intraocular pressure (IOP) is higher than 25 mmHg, topical IOP-lowering medication will be used. If IOP is higher than 40 mmHg, an intravenous drip of 20% mannitol will be used.[14] In case necessary, anterior chamber drainage will be performed through the side incision.

Outcome measures

Primary outcome

The primary outcome is the change of best corrected visual acuity (BCVA) and the incidence of complications (time frame: Non-surgical group is evaluated at the first visit and 1, 2, 3 years after enrollment. Surgical group is evaluated at preoperation, 3, 6 months, and 1, 2, 3 years postoperatively)

Secondary outcomes

1. Change of axial length (time frame: Non-surgical group is evaluated at the first visit and 1, 2, 3 years after enrollment. Surgical group is evaluated at preoperation, 3, 6 months, and 1, 2, 3 years postoperatively)

For peer review only

2. High order aberrations (time frame: Non-surgical group is evaluated at the first visit and 1, 2, 3 years after enrollment. Surgical group is evaluated at preoperation, 3, 6 months, and 1, 2, 3 years postoperatively)

3. Central cornea endothelial cell loss (time frame: Preoperation, 3, 6 months, and 1, 2, 3 years postoperatively)

4. The state of zonules (time frame: Non-surgical group is assessed at the first visit and 1, 2, 3 years after enrollment. Surgical group will be assessed preoperation)

5. Anterior chamber angle (time frame: Non-surgical group is assessed at the first visit and 1, 2, 3 years after enrollment. Surgical group will be assessed preoperation, 3, 6 months, and 1, 2, 3 years postoperatively)

6. Tilt and eccentricity of intraocular lens (time frame: 3, 6 months, and 1, 2, 3 years postoperatively)

7. Intraocular pressure (time frame: Non-surgical group is evaluated at the first visit and 1, 2, 3 years after enrollment. Surgical group is evaluated at preoperation, 3, 6 months, and 1, 2, 3 years postoperatively)

8. Aortic root diameter (time frame: Non-surgical group is evaluated at the first visit and 1, 2, 3 years after enrollment. Surgical group is evaluated at preoperation and 1, 2, 3 years postoperatively)

9. Aortic Root (Sinuses of Valsalva) Z-score, adjusted by body-surface-area (Z-score) (time frame: Non-surgical group is evaluated at the first visit and 1, 2, 3 years after enrollment. Surgical group is evaluated at preoperation and 1, 2, 3 years postoperatively)

10. Incidence of valvular heart disease (time frame: Non-surgical group is evaluated at the first visit and 1, 2, 3 years after enrollment. Surgical group is evaluated at preoperation and 1, 2, 3 years postoperatively)

11. Body mass index (BMI) (time frame: Non-surgical group is evaluated at the first visit and 1, 2, 3 years after enrollment. Surgical group is evaluated at preoperation and 1, 2, 3 years postoperatively)

12. Metacarpophalangeal joint length (time frame: Non-surgical group is evaluated at the first visit and 1, 2, 3 years after enrollment. Surgical group is evaluated at preoperation and 1, 2, 3 years postoperatively.)

13. Choroidal thickness (time frame: Non-surgical group is evaluated at the first visit and 1, 2, 3 years after enrollment. Surgical group is evaluated at preoperation and 1, 2, 3 years postoperatively)

14. Choriocapillaris flow deficits (time frame: Non-surgical group is evaluated at the first visit and 1, 2, 3 years after enrollment. Surgical group is evaluated at preoperation and 1, 2, 3 years postoperatively)

For peer review only

15. Genetic mutation state of patients (time frame: preoperation)

16. Vision-related quality of life (time frame: Non-surgical group is evaluated at the first visit and 1, 2, 3 years after enrollment. Surgical group is evaluated at preoperation and 1, 2, 3 years postoperatively)

Examinations

Baseline data and follow-up examination items are as follows:

1. Demographic characteristics, including name, gender, and date of birth.
2. Height, weight, body mass index (BMI), and length of metacarpophalangeal joints. BMI is a person's weight in kilograms divided by the square of height in meters. The normal range for the Chinese population is 18.5 to 23.9. A higher BMI can indicate higher levels of body fat. Metacarpophalangeal joint length will be measured using a palmar radiograph.
3. Echocardiography examination: This examination will be performed using doppler echocardiography (HP/Philips Sonos 5500, Philips, Bothell, WA, USA). A skilled technician will measure the aortic root diameter. Z-Score will be calculated using the Marfan foundation's calculator (https://marfan.org/dx/zscore-children/). Normal Z-score ranges from -2 to 2. A dilated aortic root is defined as a Z-score ≥ 2.0. A larger Z-score is associated with an increased risk of aortic complications such as dissection, rupture, and valvular regurgitation. An experienced cardiologist will determine the presence or absence of heart valve disease (HVD).
4. Gene detection: Genomic DNA from each subject in the study will be analyzed by whole-exome sequencing (WES) to detect mutations and diagnosis.
5. Intraocular pressure (IOP) measures: The IOP in the patient's eyes will be measured using a non-contact-tonometer at each visit, and the average of the three measures will be taken.
6. Visual acuity and subjective refraction: the uncorrected visual acuity and best corrected visual acuity (BCVA) will be evaluated with ETDRS LogMAR visual acuity chart (Precision Vision, Villa Park, Illinois, USA) at a test distance of 4.0m. The refractive error will be determined by subjective refraction following an objective measurement. Spherical equivalent (SE) will be

For peer review only

obtained with the calculation of spherical power plus half of the cylindrical power.

7. Slit-lamp examination: Slit-lamp examination (BQ-900, Haag Streit, Switzerland) and fundus examination will be performed at each visit.

8. Axial length measures: Axial length will be measured using IOLMaster 700 (Carl Zeiss AG, Jena, Germany).

8. Corneal astigmatism and high-order aberrations will be assessed using Nidek OPD-Scan III (Gamagori, Japan).

9. Corneal endothelial cell counting and morphology will be detected using an endokeratoscope (SP-2000P, Topcon, Japan).

10. The morphology of the anterior segment before surgery, particularly the state of zonules, will be assessed with ultrasound biomicroscopy (UBM).

11. The structure of the anterior chamber angle will be examined using a Tomey Casia 2 anterior segment optical coherence tomography (OCT) (Tomey, Tokyo, Japan).[15]

12. The tilt and eccentricity of IOL will be evaluated using Pentacam AXL (Oculus, Germany).[16]

13. The choroidal thickness and choriocapillaris flow deficits will be measured using posterior segment optical coherence tomography (OCT) and optical coherence tomography angiography (OCTA) (Zeiss Cirrus 5000 with AngioPlex).

14. Vision-related quality of life will be assessed using the Pediatric Eye Questionnaire (PedEyeQ).[17]

15. Adverse events or complications will be collected at each visit.

Workflow

The workflow will be carried out in the order mentioned above, starting with anthropometry (including height, weight, BMI, and length of metacarpophalangeal joints) and ending with PedEyeQ.(Figure 1 and 2). The non-surgical group will be followed up 1, 2, and 3 years after enrollment. The surgical group will be followed up at 3, 6 months, and 1, 2, 3 years postoperatively.

For peer review only

Data collection and management

The case report form (CRF) will collect basic demographic and clinical information. Results of eye examinations will be recorded on paper, and questionnaires will be filled out in the paper form. Original data will enter the lens dislocation case database at the Zhongshan Ophthalmic Center. After the study, relevant documents will be stored securely at the Zhongshan Ophthalmic Center for ten years for specific scientific research purposes.

Statistical analysis plan

Statistical analysis will be performed using Stata 15.0 (StataCorp, College Station, Texas, USA). Quantitative data conforming to a normal distribution will be described as the mean ± SD. The difference between the baseline and follow-up data will be compared by one-way analysis of variance (ANOVA). The median ± interquartile range will describe the quantitative data of the skewness distribution. The Wilcoxon signed-rank test will be used to compare these data's differences. For qualitative data described as proportions, the chi-square test or Fisher's exact test will be used to compare the differences between groups. $P<0.05$ is considered statistically significant. The differences and 95% confidence interval (CI) in the changes of BCVA and other parameters will be calculated. The univariable linear regression model will estimate these changes and their associated factors. All variables with $P<0.05$ in the univariable regression analysis will be included in the multivariable linear regression model.

Study monitoring

Clinical examiners will regularly check each patient's informed consent and eligibility to ensure that all CRFs are correct and in accordance with the original data. All errors and omissions should be recorded and corrected. The examiner should ensure every participant's withdrawal and loss of follow-up

For peer review only

are recorded and explained in CRF, and all adverse events are recorded. The surgical operations in this project are standard methods and do not pose significant risks. The principal risks include general anaesthesia adverse effects and adverse drug reactions. The trial will be conducted under the guidance of the ethics committee of the Zhongshan Ophthalmic Center.

Patient and public involvement

Neither the patients nor the public is involved in our research's design, conduct, reporting, or dissemination plans.

Ethics and dissemination

Ethics approval was obtained from the ethics committee of the Zhongshan Ophthalmic Center (number: 2022KYPJ207). Signed consents will be obtained from the legal guardians of participants after they have been informed of the study workflow and their right to withdraw from the trial. This project has been designed following the principles of the Declaration of Helsinki.

The content of this clinical study is confidential information. Clinical records and data sets will be kept at the Zhongshan Ophthalmic Center in strict confidence and will only be assessed by the study investigators and authorized personnel. The results without personal data will be disseminated through peer-reviewed publications and conference presentations.

For peer review only

Discussion

Marfan syndrome is the most common cause of congenital ectopia lentis (CEL).[18] In Germany, an average Marfan syndrome patient generates excess medical costs of 2496 euros compared to a healthy individual.[19] In China, patients with rare diseases have to cover 70% of the medical expenditure themselves.[20] Understanding different treatments' visual prognosis and complications is of great significance in reducing patients' economic burden

and improving disease management. In this study, we will measure the best corrected visual acuity (BCVA) change and the incidence of complications in patients accepting conservative and surgical treatments for CEL. In this way, we can lay the foundation for individualized management, specifically regarding the choice of surgical timing and surgical method of CEL for the future.

For peer review only

Contributors PX, KN, LJ, ZL, XZ, GJ, and DZ conceived and designed the study. PX, KN, SL, and XL wrote the draft. CY, GJ and DZ revised the draft. GJ will lead the statistical analysis. ZL, XZ, GJ and DZ will oversee data acquisition and implementation on-site. All authors reviewed and approved the final manuscript.

Funding This study was supported by the National Natural Science Foundation of China (81873673, 81900841) and the Basic and Applied Basic Research Foundation of Guangdong Province (2021A1515011673, 2022A1515011181).

Competing interests None of the authors has a financial or proprietary interest in any material or method mentioned.

Patient consent for publication Not applicable.

Provenance and peer review Not commissioned; externally peer reviewed.

For peer review only

Reference

1. Fuchs J, Rosenberg T. Congenital ectopia lentis. A Danish national survey. *Acta Ophthalmol Scand* 1998;76(1):20-26.
2. Sadiq MA, Vanderveen D. Genetics of ectopia lentis. *Semin Ophthalmol* 2013;28(5-6):313-20. doi: 10.3109/08820538.2013.825276
3. Esfandiari H, Ansari S, Mohammad-Rabei H, et al. Management Strategies of Ocular Abnormalities in Patients with Marfan Syndrome: Current Perspective. *J Ophthalmic Vis Res* 2019;14(1):71-77. doi: 10.4103/jovr.jovr_29_18
4. Rezar-Dreindl S, Stifter E, Neumayer T, et al. Visual outcome and surgical results in children with Marfan syndrome. *Clin Exp Ophthalmol* 2019;47(9):1138-45. doi: 10.1111/ceo.13596
5. Matsuo T. How far is observation allowed in patients with ectopia lentis? *Springerplus* 2015;4:461. doi: 10.1186/s40064-015-1239-5
6. Català-Mora J, Cuadras D, Díaz-Cascajosa J, et al. Anterior iris-claw intraocular lens implantation for the management of nontraumatic ectopia lentis: long-term outcomes in a paediatric cohort. *Acta Ophthalmol* 2017;95(2):170-74. doi: 10.1111/aos.13192
7. He J, Lian Z, Cao Q, et al. Longitudinal Changes of Axial Length and Associated

Factors in Congenital Ectopia Lentis Patients. *J Ophthalmol* 2022;2022:4032283. doi: 10.1155/2022/4032283

8. Hoffman RS, Snyder ME, Devgan U, et al. Management of the subluxated crystalline lens. *J Cataract Refract Surg* 2013;39(12):1904-15. doi: 10.1016/j.jcrs.2013.09.005
9. Hsu HY, Edelstein SL, Lind JT. Surgical management of non-traumatic pediatric ectopia lentis: A case series and review of the literature. *Saudi J Ophthalmol* 2012;26(3):315-21. doi: 10.1016/j.sjopt.2012.05.001
10. Stephenson KA, O'Keefe M, Keegan DJ. Surgical management of non-syndromic ectopia lentis. *International Journal of Ophthalmology* 2020;13(7):1156-60. doi: 10.18240/ijo.2020.07.21
11. Chan C-C, Liu MM, Tsai JC. The first Western-style hospital in China. *Arch Ophthalmol* 2011;129(6):791-97. doi: 10.1001/archophthalmol.2011.120
12. Chan A-W, Tetzlaff JM, Gøtzsche PC, et al. SPIRIT 2013 explanation and elaboration: guidance for protocols of clinical trials. *BMJ* 2013;346:e7586. doi: 10.1136/bmj.e7586
13. Chinese PC. Pharmacopoeia of the People's Republic of China. *China Medical Science Press: Beijing, China* 2020
14. Ramachandra V, Chandran P, Philip R, et al. Effect of Mannitol on Intraocular Pressure in Vitrectomized and Nonvitrectomized Eyes: A Prospective Comparative Study. *J Glaucoma* 2019;28(4):318-20. doi: 10.1097/IJG.0000000000001158
15. Qi H, Jin G, Zou M, et al. Characteristics of Anterior Segment in Congenital Ectopia Lentis: An SS-OCT Study. *J Ophthalmol* 2022;2022:6128832. doi: 10.1155/2022/6128832
16. Ye H, Liu Z, Cao Q, et al. Evaluation of Intraocular Lens Tilt and Decentration in Congenital Ectopia Lentis by the Pentacam Scheimpflug System. *J Ophthalmol* 2022;2022:7246730. doi: 10.1155/2022/7246730
17. Leske DA, Hatt SR, Castañeda YS, et al. Validation of the Pediatric Eye Questionnaire in Children with Visual Impairment. *Am J Ophthalmol* 2019;208:124-32. doi: 10.1016/j.ajo.2019.07.018
18. Fuchs J. Marfan syndrome and other systemic disorders with congenital ectopia lentis. A Danish national survey. *Acta Paediatr* 1997;86(9):947-52.
19. Achelrod D, Blankart CR, Linder R, et al. The economic impact of Marfan syndrome: a non-experimental, retrospective, population-based matched cohort study. *Orphanet J Rare Dis* 2014;9:90. doi: 10.1186/1750-1172-9-90

For peer review only

20. Lin J, Gong N, Cao Q, et al. What hinders congenital ectopia lentis patients' follow-up visits? A qualitative study. *BMJ Open* 2020;10(3):e030434. doi: 10.1136/bmjopen-2019-030434

For peer review only

Figure legends:

Figure 1. Flowchart of the study. CEL, congenital ectopia lentis.

Figure 2. Flowchart of the examinations. BMI, Body mass index; HVD, heart valve disease; IOP, intraocular pressure; UBM, ultrasound biomicroscopy; OCT, optical coherence tomography; IOL, intraocular lens; OCTA, optical coherence tomography angiography; PedEyeQ, Pediatric Eye Questionnaire.

For peer review only

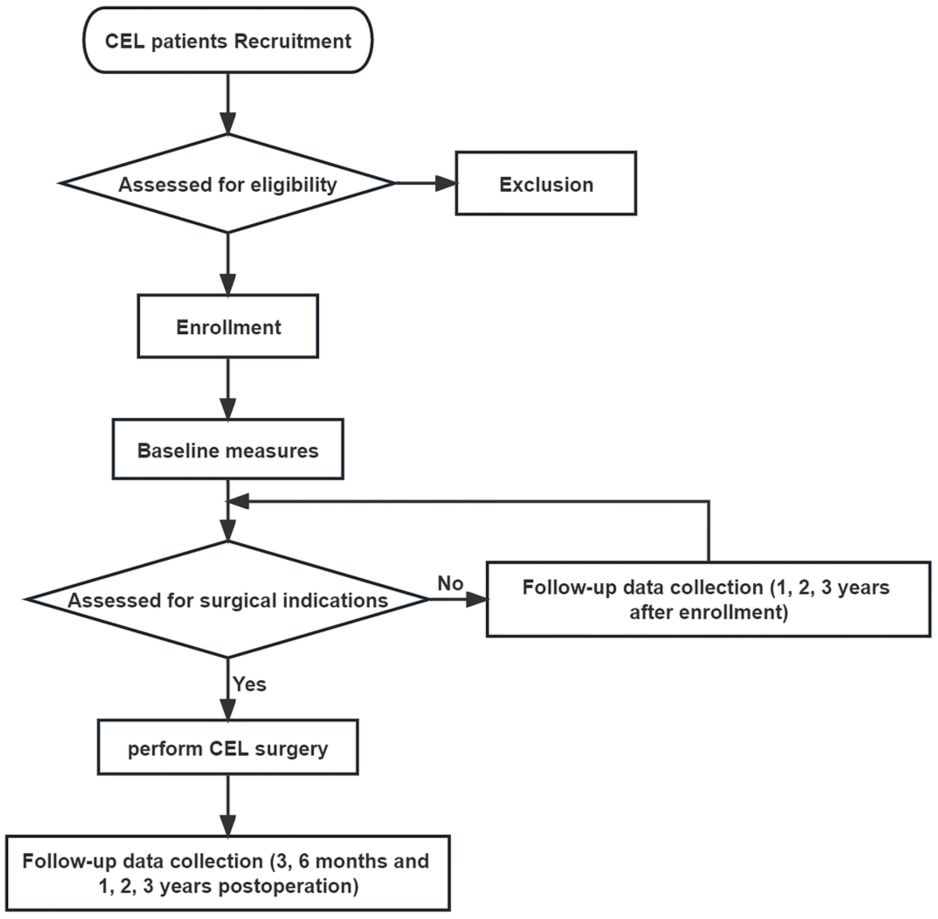

Figure 1. Flowchart of the study. CEL, congenital ectopia lentis.

455x445mm (300 x 300 DPI)

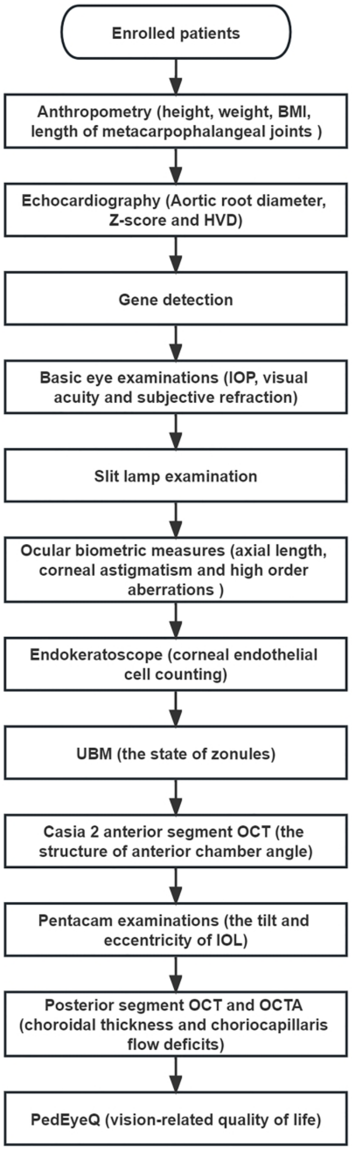

Figure 2. Flowchart of the examinations. BMI, Body mass index; HVD, heart valve disease; IOP, intraocular pressure; UBM, ultrasound biomicroscopy; OCT, optical coherence tomography; IOL, intraocular lens; OCTA, optical coherence tomography angiography; PedEyeQ, Pediatric Eye Questionnaire.

232x691mm (300 x 300 DPI)

Reporting checklist for protocol of a clinical trial.

Based on the SPIRIT guidelines.

Instructions to authors

Complete this checklist by entering the page numbers from your manuscript where readers will find each of the items listed below.

Your article may not currently address all the items on the checklist. Please modify your text to include the missing information. If you are certain that an item does not apply, please write "n/a" and provide a short explanation.

Upload your completed checklist as an extra file when you submit to a journal.

In your methods section, say that you used the SPIRIT reporting guidelines, and cite them as:

Chan A-W, Tetzlaff JM, Gøtzsche PC, Altman DG, Mann H, Berlin J, Dickersin K, Hróbjartsson A, Schulz KF, Parulekar WR, Krleža-Jerić K, Laupacis A, Moher D. SPIRIT 2013 Explanation and Elaboration: Guidance for protocols of clinical trials. BMJ. 2013;346:e7586

		Reporting Item	Page Number
**Administrative information**			
Title	#1	Descriptive title identifying the study design, population, interventions, and, if applicable, trial acronym	1
Trial registration	#2a	Trial identifier and registry name. If not yet	3,5,8-9,18

For peer review only

		registered, name of intended registry	
Trial registration: data set	#2b	All items from the World Health Organization Trial Registration Data Set	9
Protocol version	#3	Date and version identifier	n/a, 2022-Dec-27 Original
Funding	#4	Sources and types of financial, material, and other support	18
Roles and responsibilities: contributorship	#5a	Names, affiliations, and roles of protocol contributors	18
Roles and responsibilities: sponsor contact information	#5b	Name and contact information for the trial sponsor	18
Roles and responsibilities: sponsor and funder	#5c	Role of study sponsor and funders, if any, in study design; collection, management, analysis, and interpretation of data; writing of the report; and the decision to submit the report for publication, including whether they will have ultimate authority over any of these activities	n/a, study sponsor and funders don’t participate in the study
Roles and responsibilities: committees	#5d	Composition, roles, and responsibilities of the coordinating centre, steering committee, endpoint adjudication committee, data management team,	16

For peer review only

		and other individuals or groups overseeing the trial, if applicable (see Item 21a for data monitoring committee)	
**Introduction**			
Background and rationale	#6a	Description of research question and justification for undertaking the trial, including summary of relevant studies (published and unpublished) examining benefits and harms for each intervention	7
Background and rationale: choice of comparators	#6b	Explanation for choice of comparators	7
Objectives	#7	Specific objectives or hypotheses	7
Trial design	#8	Description of trial design including type of trial (eg, parallel group, crossover, factorial, single group), allocation ratio, and framework (eg, superiority, equivalence, non-inferiority, exploratory)	7
**Methods: Participants, interventions, and outcomes**			
Study setting	#9	Description of study settings (eg, community	9

For peer review only

		clinic, academic hospital) and list of countries where data will be collected. Reference to where list of study sites can be obtained	
Eligibility criteria	#10	Inclusion and exclusion criteria for participants. If applicable, eligibility criteria for study centres and individuals who will perform the interventions (eg, surgeons, psychotherapists)	8-9
Interventions: description	#11a	Interventions for each group with sufficient detail to allow replication, including how and when they will be administered	10-11
Interventions: modifications	#11b	Criteria for discontinuing or modifying allocated interventions for a given trial participant (eg, drug dose change in response to harms, participant request, or improving / worsening disease)	10
Interventions: adherance	#11c	Strategies to improve adherence to intervention protocols, and any procedures for monitoring adherence (eg, drug tablet return; laboratory tests)	9
Interventions: concomitant care	#11d	Relevant concomitant care and interventions that are permitted or prohibited during the trial	9
Outcomes	#12	Primary, secondary, and other outcomes, including the specific measurement variable (eg, systolic blood pressure), analysis metric (eg, change from baseline, final value, time to event),	11-13

For peer review only

		method of aggregation (eg, median, proportion), and time point for each outcome. Explanation of the clinical relevance of chosen efficacy and harm outcomes is strongly recommended	
Participant timeline	#13	Time schedule of enrolment, interventions (including any run-ins and washouts), assessments, and visits for participants. A schematic diagram is highly recommended (see Figure)	8
Sample size	#14	Estimated number of participants needed to achieve study objectives and how it was determined, including clinical and statistical assumptions supporting any sample size calculations	9-10
Recruitment	#15	Strategies for achieving adequate participant enrolment to reach target sample size	9
**Methods: Assignment of interventions (for controlled trials)**			
Allocation: sequence generation	#16a	Method of generating the allocation sequence (eg, computer-generated random numbers), and list of any factors for stratification. To reduce predictability of a random sequence, details of any	n/a, non-random cohort

For peer review only

		planned restriction (eg, blocking) should be provided in a separate document that is unavailable to those who enrol participants or assign interventions	
Allocation concealment mechanism	#16b	Mechanism of implementing the allocation sequence (eg, central telephone; sequentially numbered, opaque, sealed envelopes), describing any steps to conceal the sequence until interventions are assigned	n/a, non-random cohort
Allocation: implementation	#16c	Who will generate the allocation sequence, who will enrol participants, and who will assign participants to interventions	n/a, non-random cohort
Blinding (masking)	#17a	Who will be blinded after assignment to interventions (eg, trial participants, care providers, outcome assessors, data analysts), and how	n/a, un-blinded study
Blinding (masking): emergency unblinding	#17b	If blinded, circumstances under which unblinding is permissible, and procedure for revealing a participant's allocated intervention during the trial	n/a, un-blinded study
**Methods: Data collection, management, and analysis**			
Data collection plan	#18a	Plans for assessment and collection of outcome, baseline, and other trial data, including any	15

For peer review only

		related processes to promote data quality (eg, duplicate measurements, training of assessors) and a description of study instruments (eg, questionnaires, laboratory tests) along with their reliability and validity, if known. Reference to where data collection forms can be found, if not in the protocol	
Data collection plan: retention	#18b	Plans to promote participant retention and complete follow-up, including list of any outcome data to be collected for participants who discontinue or deviate from intervention protocols	15
Data management	#19	Plans for data entry, coding, security, and storage, including any related processes to promote data quality (eg, double data entry; range checks for data values). Reference to where details of data management procedures can be found, if not in the protocol	15
Statistics: outcomes	#20a	Statistical methods for analysing primary and secondary outcomes. Reference to where other details of the statistical analysis plan can be found, if not in the protocol	15
Statistics: additional analyses	#20b	Methods for any additional analyses (eg, subgroup and adjusted analyses)	15
Statistics: analysis	#20c	Definition of analysis population relating to	15

For peer review only

population and missing data		protocol non-adherence (eg, as randomised analysis), and any statistical methods to handle missing data (eg, multiple imputation)	
**Methods: Monitoring**			
Data monitoring: formal committee	#21a	Composition of data monitoring committee (DMC); summary of its role and reporting structure; statement of whether it is independent from the sponsor and competing interests; and reference to where further details about its charter can be found, if not in the protocol. Alternatively, an explanation of why a DMC is not needed	16
Data monitoring: interim analysis	#21b	Description of any interim analyses and stopping guidelines, including who will have access to these interim results and make the final decision to terminate the trial	16
Harms	#22	Plans for collecting, assessing, reporting, and managing solicited and spontaneously reported adverse events and other unintended effects of trial interventions or trial conduct	16
Auditing	#23	Frequency and procedures for auditing trial conduct, if any, and whether the process will be independent from investigators and the sponsor	16
**Ethics and dissemination**			

For peer review only

Research ethics approval	#24	Plans for seeking research ethics committee / institutional review board (REC / IRB) approval	16
Protocol amendments	#25	Plans for communicating important protocol modifications (eg, changes to eligibility criteria, outcomes, analyses) to relevant parties (eg, investigators, REC / IRBs, trial participants, trial registries, journals, regulators)	n/a, contact with the corresponding author
Consent or assent	#26a	Who will obtain informed consent or assent from potential trial participants or authorised surrogates, and how (see Item 32)	16
Consent or assent: ancillary studies	#26b	Additional consent provisions for collection and use of participant data and biological specimens in ancillary studies, if applicable	n/a
Confidentiality	#27	How personal information about potential and enrolled participants will be collected, shared, and maintained in order to protect confidentiality before, during, and after the trial	15
Declaration of interests	#28	Financial and other competing interests for principal investigators for the overall trial and each study site	18
Data access	#29	Statement of who will have access to the final trial dataset, and disclosure of contractual agreements that limit such access for investigators	15

For peer review only

Ancillary and post trial care	#30	Provisions, if any, for ancillary and post-trial care, and for compensation to those who suffer harm from trial participation	n/a
Dissemination policy: trial results	#31a	Plans for investigators and sponsor to communicate trial results to participants, healthcare professionals, the public, and other relevant groups (eg, via publication, reporting in results databases, or other data sharing arrangements), including any publication restrictions	18
Dissemination policy: authorship	#31b	Authorship eligibility guidelines and any intended use of professional writers	n/a
Dissemination policy: reproducible research	#31c	Plans, if any, for granting public access to the full protocol, participant-level dataset, and statistical code	n/a
**Appendices**			
Informed consent materials	#32	Model consent form and other related documentation given to participants and authorised surrogates	18
Biological specimens	#33	Plans for collection, laboratory evaluation, and storage of biological specimens for genetic or molecular analysis in the current trial and for future use in ancillary studies, if applicable	n/a

For peer review only

None The SPIRIT Explanation and Elaboration paper is distributed under the terms of the Creative Commons Attribution License CC-BY-NC. This checklist can be completed online using https://www.goodreports.org/, a tool made by the EQUATOR Network in collaboration with Penelope.ai

For peer review only

BMJ Open

Visual Prognosis and Complications of Congenital Ectopia Lentis: Study Protocol for a Hospital-Based Cohort in Zhongshan Ophthalmic Center

Journal:	*BMJ Open*
Manuscript ID	bmjopen-2023-072542.R1
Article Type:	Protocol
Date Submitted by the Author:	06-Jun-2023
Complete List of Authors:	Xu, Pusheng; Sun Yat-Sen University Zhongshan Ophthalmic Center State Key Laboratory of Ophthalmology<br>Ng, Kityee; Sun Yat-Sen University Zhongshan Ophthalmic Center State Key Laboratory of Ophthalmology<br>Jin, Ling; Sun Yat-Sen University Zhongshan Ophthalmic Center State Key Laboratory of Ophthalmology<br>Young, Charlotte; Albany Medical College<br>Liu, Siyuan; Sun Yat-Sen University Zhongshan Ophthalmic Center State Key Laboratory of Ophthalmology<br>Liang, Xiaolin; Sun Yat-Sen University Zhongshan Ophthalmic Center State Key Laboratory of Ophthalmology<br>Liu, Zhenzhen; Sun Yat-Sen University Zhongshan Ophthalmic Center State Key Laboratory of Ophthalmology<br>Zhang, Xinyu; Sun Yat-Sen University Zhongshan Ophthalmic Center State Key Laboratory of Ophthalmology<br>Jin, Guangming; Sun Yat-Sen University Zhongshan Ophthalmic Center State Key Laboratory of Ophthalmology<br>Zheng, Danying; Sun Yat-Sen University Zhongshan Ophthalmic Center State Key Laboratory of Ophthalmology,
**Primary Subject Heading**:	Ophthalmology
Secondary Subject Heading:	Medical management, Ophthalmology, Research methods
Keywords:	Cataract and refractive surgery < OPHTHALMOLOGY, Medical ophthalmology < OPHTHALMOLOGY, Paediatric ophthalmology < PAEDIATRIC SURGERY, Ophthalmology < SURGERY

SCHOLARONE™ Manuscripts

Visual Prognosis and Complications of Congenital Ectopia Lentis: Study Protocol for a Hospital-Based Cohort in Zhongshan Ophthalmic Center

Authors

Pusheng Xu, MD[1†]

Kityee Ng, MD[1†]

Ling Jin, MSc[1]

Charlotte Aimee Young, BS[2]

Siyuan Liu, MD[1]

Xiaolin Liang, MD[1]

Zhenzhen Liu, MD, PhD[1]

Xinyu Zhang, MD, PhD[1]

Guangming Jin, MD, PhD[1*]

Danying Zheng, MD[1*]

Institutions

1. State Key Laboratory of Ophthalmology, Zhongshan Ophthalmic Center, Sun Yat-sen University, Guangdong Provincial Key Laboratory of Ophthalmology and Visual Science, Guangdong Provincial Clinical Research Center for Ocular Diseases, Guangzhou 510060, China.

For peer review only

2. Albany Medical College, 49 New Scotland Ave Albany, New York, Unite States.

† Pusheng Xu and Kityee Ng are joint first authors.

***Corresponding Author:**

Guangming Jin, MD, PhD.

State Key Laboratory of Ophthalmology, Zhongshan Ophthalmic Center, Sun Yat-sen University, Guangdong Provincial Key Laboratory of Ophthalmology and Visual Science, Guangdong Provincial Clinical Research Center for Ocular Diseases, Guangzhou 510060, China.

Email: jingm@mail2.sysu.edu.cn

Professor Danying Zheng, MD.

State Key Laboratory of Ophthalmology, Zhongshan Ophthalmic Center, Sun Yat-sen University, Guangdong Provincial Key Laboratory of Ophthalmology and Visual Science, Guangdong Provincial Clinical Research Center for Ocular Diseases, Guangzhou 510060, China.

Email: zhengdyy@163.com

Financial support

This study was supported by the National Natural Science Foundation of China (81873673, 81900841), and the Basic and Applied Basic Research Foundation of Guangdong Province (2021A1515011673, 2022A1515011181).

For peer review only

Disclosures: None of the authors has a financial or proprietary interest in any material or method mentioned.

Number of Figures: 1

Number of Supplemental Tables: 1

Word count: 2699

For peer review only

Visual Prognosis and Complications of Congenital Ectopia Lentis: Study Protocol for a Hospital-Based Cohort in Zhongshan Ophthalmic Center

ABSTRACT

Introduction Congenital ectopia lentis (CEL) is a rare ocular disease characterized by the dislocation or displacement of the lens. Patients with mild lens dislocations can be treated with conservative methods (e.g., corrective eyeglasses or contact lenses). In contrast, patients with severe CEL usually require surgical management. However, few studies have focused on the visual prognosis and complications in conservative and surgical management patients. This study aims to investigate the prognosis and complications in CEL patients with conservative and surgical management, which is vital for CEL management, especially the choice of surgical timing and surgical method.

Methods and analysis A cohort study will be conducted at Zhongshan Ophthalmic Center. We plan to recruit 604 participants diagnosed with CEL and aged ≥ 3 years old. Patients with mild lens subluxation and stable visual conditions will be included in the non-surgical group and follow-up at 1, 2 and 3 years after enrollment. Patients with severe lens subluxation that accept CEL surgery will be included in the surgical group. Different surgical techniques, including phacoemulsification and in-the-bag intraocular lens implantation (with or without capsular tension ring), transscleral fixation, will be used depending on the severity of dislocation. Patients will be followed up at 3 months, and 1, 2 and 3 years postoperatively. Over a 5-year follow-up period, patients will receive a detailed ocular examination, including optometry, biological measurement, specular microscopy, ultrasound biomicroscopy, anterior segment and posterior segment optical coherence tomography (OCT), OCT angiography (OCTA), echocardiography and questionnaires on vision-related quality of life. The primary outcome is the change of best corrected visual acuity and the incidence of complications in both groups.

Ethics and dissemination Ethics approval was obtained from the ethics committee of the Zhongshan Ophthalmic Center (number: 2022KYPJ207). Study findings will be published in a peer-reviewed journal.

For peer review only

Trial registration number NCT05654025.

For peer review only

Strengths and limitations of this study

- This study is the first cohort of congenital ectopia lentis (CEL) in the Chinese population with a relatively large sample size and a comparatively long (5 years) follow-up.
- This study assesses important ophthalmic measurements with significant clinical implications, including visual acuity, intraocular pressure, axial length, high order aberrations, choroidal thickness and choriocapillaris flow deficits.
- Important systemic indicators of Marfan syndrome are measured, including the metacarpophalangeal joint length, incidence of valvular heart disease, aortic root diameter and Z-score.
- In this study, CEL in age less than 3 years old are excluded and participants could be lost to follow-up after surgery.
- This study focuses on individuals with CEL but different subtypes of CEL may not be classified.

For peer review only

INTRODUCTION

Congenital ectopia lentis (CEL) is a rare ocular disease characterized by the dislocation or displacement of the lens. Several systemic diseases have been reported to be associated with CEL, such as Marfan syndrome, homocystinuria, Weill-Marchesani syndrome and Ehlers-Danlos syndrome.[1] However, some patients with CEL may have no known systemic manifestations.[2]

Patients with mild lens dislocations can be treated with conservative methods (e.g., corrective eyeglasses or contact lenses).[3] However, progressive subluxation or complete dislocation of the lens can cause a high degree of myopia or astigmatism, even amblyopia.[4] Surgical intervention is crucial in the management of patients with severe CEL.[5] Currently, there is no unified standard for operation timing for CEL. A previous study recommended surgical intervention when the near vision of children was less than 0.4 in LogMAR acuity.[6] However, some researchers suggested that surgery should be performed when the best corrected visual acuity (BCVA) is less than 0.3 or monocular diplopia occurs.[4 7] Few studies have focused on the refractive change and visual prognosis in CEL patients both with conservative management and surgical management.[8]

Several surgical techniques have been reported over the past decades, such as lensectomy, phacoemulsification without intraocular lens (IOL) implantation, phacoemulsification and IOL implantation (with or without capsular tension ring) and various transscleral fixation of IOL.[9-11] However, the safety and efficacy of these techniques have not been fully validated so far, especially in the Chinese population. Herein we will conduct this cohort study at the Zhongshan Ophthalmic Center, one of the biggest ophthalmic hospitals in China.[12] All children diagnosed with CEL will be followed up for at least three years. Long-term changes in best-corrected visual acuity and the incidence of complications will be evaluated in patients with conservative management and surgical management.

METHODS AND ANALYSIS

For peer review only

This study will be conducted from 5 December 2022 to 5 December 2027. The study was registered on Clinicaltrials.gov (NCT05654025).

Objective

The study is designed as a prospective clinical trial to investigate the prognosis and complications in CEL patients with conservative and surgical management. The aim is to serve as a reference for disease management, specifically for choice of surgical timing and surgical method.

Study design

An approximately 5-year cohort study will be conducted at Zhongshan Ophthalmic Centre, Guangzhou, China. This study will adhere to the Declaration of Helsinki, and ethics have been approved by the ethics committee of the Zhongshan Ophthalmic Center (number: 2022KYPJ207). This study is designed used the SPIRIT reporting guidelines.[13]

Eligibility criteria

Inclusion criteria

1. Diagnosed with congenital lens dislocation and age ≥ 3 years old.

2. Agree to participate in this study with written informed consent from patients or legal guardians.

Exclusion Criteria

1. History of ophthalmic trauma or other ophthalmic surgeries.

2. Combined with other ophthalmic diseases such as primary glaucoma, uveitis and corneal disease.

3. Patients who could not cooperate in the examinations.

For peer review only

Study setting and participants

This cohort study will be conducted at Zhongshan Ophthalmic Center, one of the largest ophthalmic hospitals in China. This study aims to investigate the visual prognosis and complications in CEL patients with different managements. Patients aged three years or above diagnosed with CEL will be recruited from Zhongshan Ophthalmic Center. Moreover, each participant will be followed for at least three years.

Once the participants meet the requirement of our eligibility criteria, they will be asked to join the WeChat (an instant messaging tool) group on the phone and be provided with informed consent. Interested participants or their guardians will sign the consent form and, if appropriate, will complete a thorough ocular examination and systemic evaluations.

Recruitment

The outpatient clinics will carry out the first screening at Zhongshan Ophthalmic Center. Potential participants will be further confirmed by eligibility and recruited at Zhongshan Ophthalmic Center for clinical trials. One of our researchers will contact the participants and explain the trial process in detail to ensure the participants or guardians fully understand the whole study. Once they agree and sign the consent form, further information will be provided, including the purpose of the study, examinations, the importance of follow-up time and duration, and possible risk in treatment. Then the trial will proceed subsequently.

Sample size

Sample size will be estimated as follows: Assuming the incidence of complications in CEL patients is 15%, and the margin of error is 20%. For a 5% significance level, $Z_{\alpha/2}$ is 1.96 for the two-tailed alternative hypothesis. Sample size = $(Z_{\alpha/2})^2 \cdot P(1-P) * 1/E^2 = (1.96)^2 \cdot 15\% \cdot (1-15\%)/(15\% \cdot 20\%) = 544$. Assuming the loss ratio of 10%, the adjusted sample size will be 544/(1-0.1) = 544/0.9=

For peer review only - http://bmjopen.bmj.com/site/about/guidelines.xhtml

604.

Preoperative management

All patients diagnosed with CEL will be followed up since the enrollment. The surgery will be performed when either of the following situations occurs: 1) BCVA < 0.3 in LogMAR acuity; 2) monocular diplopia; 3) progressive subluxation of the lens affecting the pupillary axis; 4) complicated with severe cataract or secondary glaucoma or corneal endothelial decompensation or retinal detachment.[8]

Preoperative medication

All patients will be routinely administered levofloxacin or tobramycin eye drops (four times a day for three days) before surgery to minimize the risk of infection. An intramuscular injection of ethamsylate (1mg/kg)[14] will be used 30 minutes before surgery to reduce bleeding.

Anaesthesia

General anaesthesia or retrobulbar anaesthesia will be used according to the standard clinical routine.

Surgery methods management

The choice of surgical methods depends on the degree of ectopia lentis and the state of zonules. If the extent of the unhealthy zonules (broken or weak) ≤ 180°, phacoemulsification and in-the-bag IOL implantation (with or without capsular tension ring) will be used. Otherwise, the capsular bag will be removed, and IOL will be fixed through transscleral fixation. Surgery will be performed by the same surgeon (Dr. DY Zheng). Rayner 920H/970C or Sensar AR40e will be used as the implanted IOL.

The surgical techniques for patients who received in-the-bag IOL

For peer review only

implantation are as follows: A 3.0 mm corneal tunnel incision will be made at 12 o'clock. Then, a continuous circular capsulorhexis will be performed manually. Iris hooks will stabilize the bag, and the lens will be aspirated with a phacoemulsifer. IOL will be implanted in-the-bag, and the capsular tension ring will be implanted when the IOL cannot be stably fixed.

For patients who received transscleral fixation of IOL, the surgical techniques will be performed as the previous study described.[8] In brief, a 3.0 mm corneal tunnel incision will be made at 12 o'clock. Then, a continuous circular capsulorhexis will be performed manually, and the capsular bag will be removed after the phacoaspiration. Transscleral fixation of IOL will be performed with the two IOL haptic sutured by 8-0 polypropylene at 2.0 mm posterior to the corneal limbus. Anterior vitrectomy will be performed when severe vitreous prolapse occurs.

For peer review only

Postoperative management

After surgery, tobramycin and dexamethasone eye drops (four times a day) and ointment (once every night) will be routinely administered for one week. If intraocular pressure (IOP) is higher than 25 mmHg, topical IOP-lowering medication will be used. If IOP is higher than 40 mmHg, an intravenous drip of 20% mannitol will be used.[15] In case necessary, anterior chamber drainage will be performed through the side incision.

Outcome measures

Primary outcome

The primary outcome is the change of best corrected visual acuity (BCVA) and the incidence of complications (time frame: Non-surgical group is evaluated at the first visit and 1, 2, 3 years after enrollment. Surgical group is evaluated at preoperation, 3 months, and 1, 2, 3 years postoperatively)

Secondary outcomes

1. Change of axial length (time frame: Non-surgical group is evaluated at the

first visit and 1, 2, 3 years after enrollment. Surgical group is evaluated at preoperation, 3 months, and 1, 2, 3 years postoperatively)

2. High order aberrations (time frame: Non-surgical group is evaluated at the first visit and 1, 2, 3 years after enrollment. Surgical group is evaluated at preoperation, 3 months, and 1, 2, 3 years postoperatively)

3. Central cornea endothelial cell loss (time frame: Preoperation, 3 months, and 1, 2, 3 years postoperatively)

4. The state of zonules (time frame: Non-surgical group is assessed at the first visit and 1, 2, 3 years after enrollment. Surgical group will be assessed preoperation)

5. Anterior chamber angle (time frame: Non-surgical group is assessed at the first visit and 1, 2, 3 years after enrollment. Surgical group will be assessed preoperation, 3months, and 1, 2, 3 years postoperatively)

6. Tilt and eccentricity of intraocular lens (time frame: 3 months, and 1, 2, 3 years postoperatively)

7. Intraocular pressure (time frame: Non-surgical group is evaluated at the first visit and 1, 2, 3 years after enrollment. Surgical group is evaluated at preoperation, 3 months, and 1, 2, 3 years postoperatively)

8. Aortic root diameter (time frame: Non-surgical group is evaluated at the first visit and 1, 2, 3 years after enrollment. Surgical group is evaluated at preoperation and 1, 2, 3 years postoperatively)

9. Aortic Root (Sinuses of Valsalva) Z-score, adjusted by body-surface-area (Z-score) (time frame: Non-surgical group is evaluated at the first visit and 1, 2, 3 years after enrollment. Surgical group is evaluated at preoperation and 1, 2, 3 years postoperatively)

10. Incidence of valvular heart disease (time frame: Non-surgical group is evaluated at the first visit and 1, 2, 3 years after enrollment. Surgical group is evaluated at preoperation and 1, 2, 3 years postoperatively)

11. Body mass index (BMI) (time frame: Non-surgical group is evaluated at the first visit and 1, 2, 3 years after enrollment. Surgical group is evaluated at preoperation and 1, 2, 3 years postoperatively)

12. Metacarpophalangeal joint length (time frame: Non-surgical group is evaluated at the first visit and 1, 2, 3 years after enrollment. Surgical group is evaluated at preoperation and 1, 2, 3 years postoperatively.)

13. Choroidal thickness (time frame: Non-surgical group is evaluated at the first visit and 3 years after enrollment. Surgical group is evaluated at preoperation and 3 years postoperatively)

14. Choriocapillaris flow deficits (time frame: Non-surgical group is evaluated

For peer review only

at the first visit and 3 years after enrollment. Surgical group is evaluated at preoperation and 3 years postoperatively)

15. Genetic mutation state of patients (time frame: preoperation)

16. Vision-related quality of life (time frame: Non-surgical group is evaluated at the first visit and 3 years after enrollment. Surgical group is evaluated at preoperation and 3 years postoperatively)

Examinations

Baseline data and follow-up examination items are as follows:

1. Demographic characteristics, including name, gender, and date of birth.
2. Slit-lamp examination: Slit-lamp examination (BQ-900, Haag Streit, Switzerland) and fundus examination will be performed at each visit.
3. Visual acuity and refraction: the uncorrected visual acuity and best corrected visual acuity (BCVA) will be evaluated with ETDRS LogMAR visual acuity chart (Precision Vision, Villa Park, Illinois, USA) at a test distance of 4.0m. The refractive error will be determined by subjective refraction following an objective measurement. Spherical equivalent (SE) will be obtained with the calculation of spherical power plus half of the cylindrical power.
4. Axial length measures: Axial length will be measured using IOLMaster 700 (Carl Zeiss AG, Jena, Germany).
5. High-order aberrations will be assessed using Nidek OPD-Scan III (Gamagori, Japan).
6. Corneal endothelial cell counting and morphology will be detected using an endokeratoscope (SP-2000P, Topcon, Japan).
7. The state of zonules will be assessed clock by clock with ultrasound biomicroscopy (UBM). The clock range of zonular disruption or loosening will be recorded. And this results will be confirmed using slit-lamp photography after dilation.
8. The structure of the anterior chamber angle will be examined using a Tomey Casia 2 anterior segment optical coherence tomography (OCT) (Tomey, Tokyo, Japan).[16]
9. The tilt and eccentricity of IOL will be evaluated using Pentacam AXL (Oculus, Germany).[17]

For peer review only

10. Intraocular pressure (IOP) measures: The IOP in the patient's eyes will be measured using a non-contact-tonometer at each visit, and the average of the three measures will be taken.

11. Echocardiography examination: This examination will be performed using doppler echocardiography (HP/Philips Sonos 5500, Philips, Bothell, WA, USA). A skilled technician will measure the aortic root diameter. Z-Score will be calculated using the Marfan foundation's calculator (https://marfan.org/dx/zscore-children/). Normal Z-score ranges from -2 to 2. A dilated aortic root is defined as a Z-score ≥ 2.0. A larger Z-score is associated with an increased risk of aortic complications such as dissection, rupture, and valvular regurgitation. An experienced cardiologist will determine the presence or absence of heart valve disease (HVD).

12. Body mass index (BMI). BMI is a person's weight in kilograms divided by the square of height in meters. The normal range for the Chinese population is 18.5 to 23.9. A higher BMI can indicate higher levels of body fat.

13. Metacarpophalangeal joint length will be measured in hand radiograph.

14. The choroidal thickness and choriocapillaris flow deficits will be measured using posterior segment optical coherence tomography (OCT) and optical coherence tomography angiography (OCTA) (Zeiss Cirrus 5000 with AngioPlex).

15. Gene detection: Genomic DNA from each subject in the study will be analyzed by whole-exome sequencing (WES) to detect mutations and diagnosis.

16. Vision-related quality of life will be assessed using the Pediatric Eye Questionnaire (PedEyeQ).[18]

17. Adverse events or complications will be collected at each visit.

Workflow

The workflow will be carried out in the order mentioned above, starting with patients recruitment and ending with a minimum of 3 years follow-up. (Figure 1). The non-surgical group will be followed up 1, 2, and 3 years after enrollment. The surgical group will be followed up at 3 months, and 1, 2, 3 years postoperatively. The examinations planned for each follow-up time point are

For peer review only

shown in Supplemental table 1.

Data collection and management

The case report form (CRF) will collect basic demographic and clinical information. Results of eye examinations will be recorded on paper, and questionnaires will be filled out in the paper form. Original data will enter the lens dislocation case database at the Zhongshan Ophthalmic Center. After the study, relevant documents will be stored securely at the Zhongshan Ophthalmic Center for ten years for specific scientific research purposes.

Statistical analysis plan

Statistical analysis will be performed using Stata 15.0 (StataCorp, College Station, Texas, USA). Quantitative data conforming to a normal distribution will be described as the mean ± SD. The difference between the baseline and follow-up data will be compared by one-way analysis of variance (ANOVA). The median ± interquartile range will describe the quantitative data of the skewness distribution. The Wilcoxon signed-rank test will be used to compare these data's differences. For qualitative data described as proportions, the chi-square test or Fisher's exact test will be used to compare the differences between groups. $P<0.05$ is considered statistically significant. The differences and 95% confidence interval (CI) in the changes of BCVA and other parameters will be calculated. The univariable linear regression model will estimate these changes and their associated factors. All variables with $P<0.05$ in the univariable regression analysis will be included in the multivariable linear regression model.

Study monitoring

Clinical examiners will regularly check each patient's informed consent and eligibility to ensure that all CRFs are correct and in accordance with the original data. All errors and omissions should be recorded and corrected. The examiner should ensure every participant's withdrawal and loss of follow-up are recorded and explained in CRF, and all adverse events are recorded. The surgical operations in this project are standard methods and do not pose significant risks.

For peer review only

The principal risks include general anaesthesia adverse effects and adverse drug reactions. This cohort study will be conducted under the guidance of the ethics committee of the Zhongshan Ophthalmic Center.

Patient and public involvement

Neither the patients nor the public is involved in our research's design, conduct, reporting, or dissemination plans.

Ethics and dissemination

Ethics approval was obtained from the ethics committee of the Zhongshan Ophthalmic Center (number: 2022KYPJ207). Signed consents will be obtained from the legal guardians of participants after they have been informed of the study workflow and their right to withdraw from the cohort study. This project has been designed following the principles of the Declaration of Helsinki.

The content of this cohort study is confidential information. Clinical records and data sets will be kept at the Zhongshan Ophthalmic Center in strict confidence and will only be assessed by the study investigators and authorized personnel. The results without personal data will be disseminated through peer-reviewed publications and conference presentations.

For peer review only

Contributors PX, KN, LJ, ZL, XZ, GJ, and DZ conceived and designed the study. PX, KN, SL, and XL wrote the draft. CY, GJ and DZ revised the draft. GJ will lead the statistical analysis. ZL, XZ, GJ and DZ will oversee data acquisition and implementation on-site. All authors reviewed and approved the final manuscript.

Funding This study was supported by the National Natural Science Foundation of China (81873673, 81900841) and the Basic and Applied Basic Research Foundation of Guangdong Province (2021A1515011673, 2022A1515011181).

Competing interests None of the authors has a financial or proprietary interest in any material or method mentioned.

Data Availability Statement Data are available upon reasonable request

Patient consent for publication Not applicable.

Provenance and peer review Not commissioned; externally peer reviewed.

Reference

1. Sadiq MA, Vanderveen D. Genetics of ectopia lentis. *Semin Ophthalmol* 2013;28(5-6):313-20. doi: 10.3109/08820538.2013.825276
2. Guo D, Yang F, Zhou Y, et al. Novel ADAMTSL4 gene mutations in Chinese patients with isolated ectopia lentis. *Br J Ophthalmol* 2022 doi: 10.1136/bjophthalmol-2021-320475
3. Esfandiari H, Ansari S, Mohammad-Rabei H, et al. Management Strategies of Ocular Abnormalities in Patients with Marfan Syndrome: Current Perspective. *J Ophthalmic Vis Res* 2019;14(1):71-77. doi: 10.4103/jovr.jovr_29_18
4. Rezar-Dreindl S, Stifter E, Neumayer T, et al. Visual outcome and surgical results in children with Marfan syndrome. *Clin Exp Ophthalmol* 2019;47(9):1138-45. doi: 10.1111/ceo.13596
5. Chee S-P, Ti S-E, Chan NS-W. Management of the subluxated crystalline lens: A review. *Clin Exp Ophthalmol* 2021;49(9):1091-101. doi: 10.1111/ceo.13975
6. Matsuo T. How far is observation allowed in patients with ectopia lentis? *Springerplus* 2015;4:461. doi: 10.1186/s40064-015-1239-5
7. Català-Mora J, Cuadras D, Díaz-Cascajosa J, et al. Anterior iris-claw intraocular lens implantation for the management of nontraumatic ectopia lentis: long-term outcomes in a paediatric cohort. *Acta Ophthalmol* 2017;95(2):170-74. doi: 10.1111/aos.13192
8. He J, Lian Z, Cao Q, et al. Longitudinal Changes of Axial Length and Associated Factors in Congenital Ectopia Lentis Patients. *J Ophthalmol* 2022;2022:4032283. doi: 10.1155/2022/4032283
9. Hoffman RS, Snyder ME, Devgan U, et al. Management of the subluxated crystalline lens. *J Cataract Refract Surg* 2013;39(12):1904-15. doi: 10.1016/j.jcrs.2013.09.005

10. Hsu HY, Edelstein SL, Lind JT. Surgical management of non-traumatic pediatric ectopia lentis: A case series and review of the literature. *Saudi J Ophthalmol* 2012;26(3):315-21. doi: 10.1016/j.sjopt.2012.05.001
11. Stephenson KA, O'Keefe M, Keegan DJ. Surgical management of non-syndromic ectopia lentis. *International Journal of Ophthalmology* 2020;13(7):1156-60. doi: 10.18240/ijo.2020.07.21
12. Chan C-C, Liu MM, Tsai JC. The first Western-style hospital in China. *Arch Ophthalmol* 2011;129(6):791-97. doi: 10.1001/archophthalmol.2011.120
13. Chan A-W, Tetzlaff JM, Gøtzsche PC, et al. SPIRIT 2013 explanation and elaboration: guidance for protocols of clinical trials. *BMJ* 2013;346:e7586. doi: 10.1136/bmj.e7586
14. Chinese PC. Pharmacopoeia of the People’s Republic of China. *China Medical Science Press: Beijing, China* 2020
15. Ramachandra V, Chandran P, Philip R, et al. Effect of Mannitol on Intraocular Pressure in Vitrectomized and Nonvitrectomized Eyes: A Prospective Comparative Study. *J Glaucoma* 2019;28(4):318-20. doi: 10.1097/IJG.0000000000001158
16. Qi H, Jin G, Zou M, et al. Characteristics of Anterior Segment in Congenital Ectopia Lentis: An SS-OCT Study. *J Ophthalmol* 2022;2022:6128832. doi: 10.1155/2022/6128832
17. Ye H, Liu Z, Cao Q, et al. Evaluation of Intraocular Lens Tilt and Decentration in Congenital Ectopia Lentis by the Pentacam Scheimpflug System. *J Ophthalmol* 2022;2022:7246730. doi: 10.1155/2022/7246730
18. Leske DA, Hatt SR, Castañeda YS, et al. Validation of the Pediatric Eye Questionnaire in Children with Visual Impairment. *Am J Ophthalmol* 2019;208:124-32. doi: 10.1016/j.ajo.2019.07.018

For peer review only

Figure legends:

Figure 1. Flowchart of the study. CEL, congenital ectopia lentis. * The surgery will be performed when either of the following situations occurs.

For peer review only

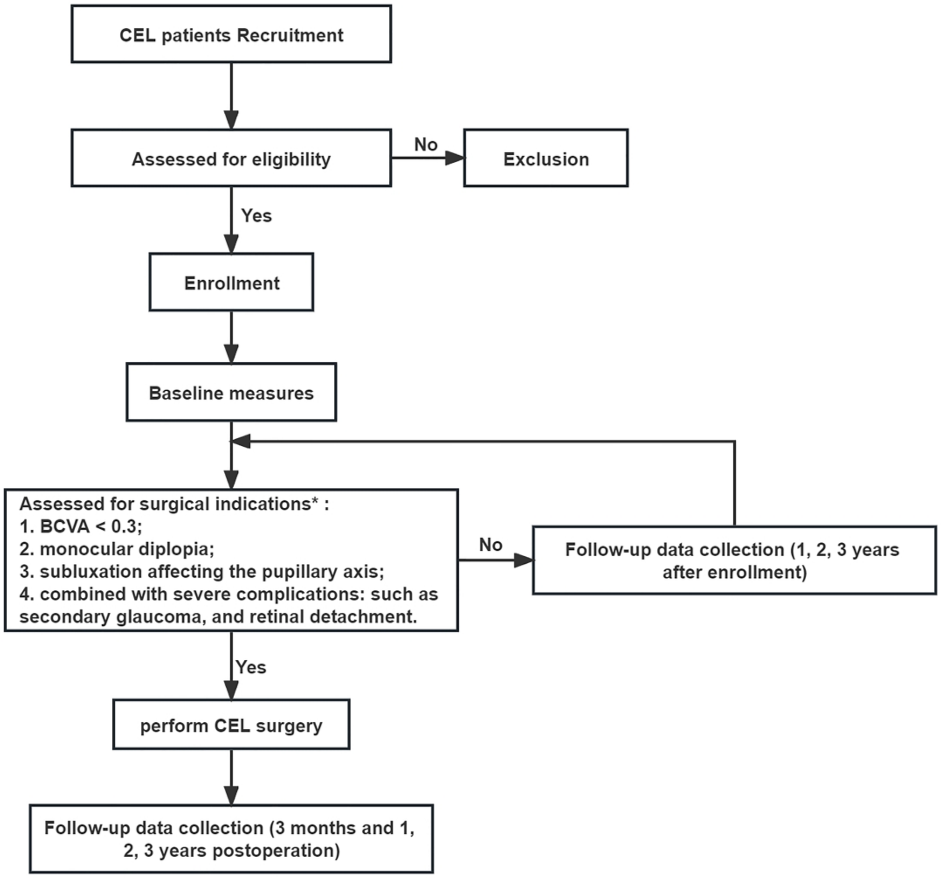

Figure 1. Flowchart of the study. CEL, congenital ectopia lentis. * The surgery will be performed when either of the following situations occurs.

500x469mm (300 x 300 DPI)

Supplemental table 1. Complete overview of follow-up and examinations.

Examination	Time points of non-surgical group				Time points of surgical group				
	Baseline	1 y	2 y	3 y	Preoperation*	3 m	1 y	2 y	3 y
Demographic data	✓				✓				
Slit-lamp examination	✓	✓	✓	✓	✓	✓	✓	✓	✓
Visual acuity and refraction	✓	✓	✓	✓	✓	✓	✓	✓	✓
Axial length	✓	✓	✓	✓	✓	✓	✓	✓	✓
High-order aberrations	✓	✓	✓	✓	✓	✓	✓	✓	✓
Specular microscopy					✓	✓	✓	✓	✓
Anterior segment OCT	✓	✓	✓	✓	✓	✓	✓	✓	✓
UBM	✓	✓	✓	✓	✓				
Slit-lamp photography	✓	✓	✓	✓	✓				
Pentacam	✓	✓	✓	✓	✓	✓	✓	✓	✓
IOP	✓	✓	✓	✓	✓	✓	✓	✓	✓
Echocardiography	✓	✓	✓	✓	✓		✓	✓	✓
BMI	✓	✓	✓	✓	✓		✓	✓	✓
Hand radiograph	✓	✓	✓	✓	✓		✓	✓	✓
Posterior segment OCT	✓			✓	✓				✓
Gene detection†	✓				✓				
PedEyeQ	✓			✓	✓				✓
Adverse events	✓	✓	✓	✓	✓	✓	✓	✓	✓

* Preoperation examninations will not be repeated if the patient has just completed follow-up and meets the surgical indications.

† Gene detection will not be repeated if the patient already has a valid genetic testing report.

OCT, optical coherence tomography; UBM, ultrasound biomicroscopy; IOP, intraocular pressure; BMI, body mass index; PedEyeQ, the Pediatric Eye Questionnaire; y, years; m, months.

For peer review only

Reporting checklist for protocol of a clinical trial.

Based on the SPIRIT guidelines.

Instructions to authors

Complete this checklist by entering the page numbers from your manuscript where readers will find each of the items listed below.

Your article may not currently address all the items on the checklist. Please modify your text to include the missing information. If you are certain that an item does not apply, please write "n/a" and provide a short explanation.

Upload your completed checklist as an extra file when you submit to a journal.

In your methods section, say that you used the SPIRIT reporting guidelines, and cite them as:

Chan A-W, Tetzlaff JM, Gøtzsche PC, Altman DG, Mann H, Berlin J, Dickersin K, Hróbjartsson A, Schulz KF, Parulekar WR, Krleža-Jerić K, Laupacis A, Moher D. SPIRIT 2013 Explanation and Elaboration: Guidance for protocols of clinical trials. BMJ. 2013;346:e7586

For peer review only

		Reporting Item	Page Number
**Administrative information**			
Title	#1	Descriptive title identifying the study design, population, interventions, and, if applicable, trial acronym	1
Trial registration	#2a	Trial identifier and registry name. If not yet	3,5,8-9,18

		registered, name of intended registry	
Trial registration: data set	#2b	All items from the World Health Organization Trial Registration Data Set	9
Protocol version	#3	Date and version identifier	n/a, 2022-Dec-27 Original
Funding	#4	Sources and types of financial, material, and other support	18
Roles and responsibilities: contributorship	#5a	Names, affiliations, and roles of protocol contributors	18
Roles and responsibilities: sponsor contact information	#5b	Name and contact information for the trial sponsor	18
Roles and responsibilities: sponsor and funder	#5c	Role of study sponsor and funders, if any, in study design; collection, management, analysis, and interpretation of data; writing of the report; and the decision to submit the report for publication, including whether they will have ultimate authority over any of these activities	n/a, study sponsor and funders don't participate in the study
Roles and responsibilities: committees	#5d	Composition, roles, and responsibilities of the coordinating centre, steering committee, endpoint adjudication committee, data management team,	16

For peer review only

		and other individuals or groups overseeing the trial, if applicable (see Item 21a for data monitoring committee)	
**Introduction**			
Background and rationale	#6a	Description of research question and justification for undertaking the trial, including summary of relevant studies (published and unpublished) examining benefits and harms for each intervention	7
Background and rationale: choice of comparators	#6b	Explanation for choice of comparators	7
Objectives	#7	Specific objectives or hypotheses	7
Trial design	#8	Description of trial design including type of trial (eg, parallel group, crossover, factorial, single group), allocation ratio, and framework (eg, superiority, equivalence, non-inferiority, exploratory)	7
**Methods: Participants, interventions, and outcomes**			
Study setting	#9	Description of study settings (eg, community	9

For peer review only

		clinic, academic hospital) and list of countries where data will be collected. Reference to where list of study sites can be obtained	
Eligibility criteria	#10	Inclusion and exclusion criteria for participants. If applicable, eligibility criteria for study centres and individuals who will perform the interventions (eg, surgeons, psychotherapists)	8-9
Interventions: description	#11a	Interventions for each group with sufficient detail to allow replication, including how and when they will be administered	10-11
Interventions: modifications	#11b	Criteria for discontinuing or modifying allocated interventions for a given trial participant (eg, drug dose change in response to harms, participant request, or improving / worsening disease)	10
Interventions: adherance	#11c	Strategies to improve adherence to intervention protocols, and any procedures for monitoring adherence (eg, drug tablet return; laboratory tests)	9
Interventions: concomitant care	#11d	Relevant concomitant care and interventions that are permitted or prohibited during the trial	9
Outcomes	#12	Primary, secondary, and other outcomes, including the specific measurement variable (eg, systolic blood pressure), analysis metric (eg, change from baseline, final value, time to event),	11-13

For peer review only

		method of aggregation (eg, median, proportion), and time point for each outcome. Explanation of the clinical relevance of chosen efficacy and harm outcomes is strongly recommended	
Participant timeline	#13	Time schedule of enrolment, interventions (including any run-ins and washouts), assessments, and visits for participants. A schematic diagram is highly recommended (see Figure)	8
Sample size	#14	Estimated number of participants needed to achieve study objectives and how it was determined, including clinical and statistical assumptions supporting any sample size calculations	9-10
Recruitment	#15	Strategies for achieving adequate participant enrolment to reach target sample size	9
**Methods: Assignment of interventions (for controlled trials)**			
Allocation: sequence generation	#16a	Method of generating the allocation sequence (eg, computer-generated random numbers), and list of any factors for stratification. To reduce predictability of a random sequence, details of any	n/a, non-random cohort

For peer review only

		planned restriction (eg, blocking) should be provided in a separate document that is unavailable to those who enrol participants or assign interventions	
Allocation concealment mechanism	#16b	Mechanism of implementing the allocation sequence (eg, central telephone; sequentially numbered, opaque, sealed envelopes), describing any steps to conceal the sequence until interventions are assigned	n/a, non-random cohort
Allocation: implementation	#16c	Who will generate the allocation sequence, who will enrol participants, and who will assign participants to interventions	n/a, non-random cohort
Blinding (masking)	#17a	Who will be blinded after assignment to interventions (eg, trial participants, care providers, outcome assessors, data analysts), and how	n/a, un-blinded study
Blinding (masking): emergency unblinding	#17b	If blinded, circumstances under which unblinding is permissible, and procedure for revealing a participant's allocated intervention during the trial	n/a, un-blinded study
**Methods: Data collection, management, and analysis**			
Data collection plan	#18a	Plans for assessment and collection of outcome, baseline, and other trial data, including any	15

For peer review only

		related processes to promote data quality (eg, duplicate measurements, training of assessors) and a description of study instruments (eg, questionnaires, laboratory tests) along with their reliability and validity, if known. Reference to where data collection forms can be found, if not in the protocol	
Data collection plan: retention	#18b	Plans to promote participant retention and complete follow-up, including list of any outcome data to be collected for participants who discontinue or deviate from intervention protocols	15
Data management	#19	Plans for data entry, coding, security, and storage, including any related processes to promote data quality (eg, double data entry; range checks for data values). Reference to where details of data management procedures can be found, if not in the protocol	15
Statistics: outcomes	#20a	Statistical methods for analysing primary and secondary outcomes. Reference to where other details of the statistical analysis plan can be found, if not in the protocol	15
Statistics: additional analyses	#20b	Methods for any additional analyses (eg, subgroup and adjusted analyses)	15
Statistics: analysis	#20c	Definition of analysis population relating to	15

For peer review only

population and missing data		protocol non-adherence (eg, as randomised analysis), and any statistical methods to handle missing data (eg, multiple imputation)	
**Methods: Monitoring**			
Data monitoring: formal committee	#21a	Composition of data monitoring committee (DMC); summary of its role and reporting structure; statement of whether it is independent from the sponsor and competing interests; and reference to where further details about its charter can be found, if not in the protocol. Alternatively, an explanation of why a DMC is not needed	16
Data monitoring: interim analysis	#21b	Description of any interim analyses and stopping guidelines, including who will have access to these interim results and make the final decision to terminate the trial	16
Harms	#22	Plans for collecting, assessing, reporting, and managing solicited and spontaneously reported adverse events and other unintended effects of trial interventions or trial conduct	16
Auditing	#23	Frequency and procedures for auditing trial conduct, if any, and whether the process will be independent from investigators and the sponsor	16
**Ethics and dissemination**			

For peer review only

Research ethics approval	#24	Plans for seeking research ethics committee / institutional review board (REC / IRB) approval	16
Protocol amendments	#25	Plans for communicating important protocol modifications (eg, changes to eligibility criteria, outcomes, analyses) to relevant parties (eg, investigators, REC / IRBs, trial participants, trial registries, journals, regulators)	n/a, contact with the corresponding author
Consent or assent	#26a	Who will obtain informed consent or assent from potential trial participants or authorised surrogates, and how (see Item 32)	16
Consent or assent: ancillary studies	#26b	Additional consent provisions for collection and use of participant data and biological specimens in ancillary studies, if applicable	n/a
Confidentiality	#27	How personal information about potential and enrolled participants will be collected, shared, and maintained in order to protect confidentiality before, during, and after the trial	15
Declaration of interests	#28	Financial and other competing interests for principal investigators for the overall trial and each study site	18
Data access	#29	Statement of who will have access to the final trial dataset, and disclosure of contractual agreements that limit such access for investigators	15

For peer review only

Ancillary and post trial care	#30	Provisions, if any, for ancillary and post-trial care, and for compensation to those who suffer harm from trial participation	n/a
Dissemination policy: trial results	#31a	Plans for investigators and sponsor to communicate trial results to participants, healthcare professionals, the public, and other relevant groups (eg, via publication, reporting in results databases, or other data sharing arrangements), including any publication restrictions	18
Dissemination policy: authorship	#31b	Authorship eligibility guidelines and any intended use of professional writers	n/a
Dissemination policy: reproducible research	#31c	Plans, if any, for granting public access to the full protocol, participant-level dataset, and statistical code	n/a

Appendices

Informed consent materials	#32	Model consent form and other related documentation given to participants and authorised surrogates	18
Biological specimens	#33	Plans for collection, laboratory evaluation, and storage of biological specimens for genetic or molecular analysis in the current trial and for future use in ancillary studies, if applicable	n/a

For peer review only

None The SPIRIT Explanation and Elaboration paper is distributed under the terms of the Creative Commons Attribution License CC-BY-NC. This checklist can be completed online using https://www.goodreports.org/, a tool made by the EQUATOR Network in collaboration with Penelope.ai

For peer review only

BMJ Open

Visual Prognosis and Complications of Congenital Ectopia Lentis: Study Protocol for a Hospital-Based Cohort in Zhongshan Ophthalmic Center

Journal:	*BMJ Open*
Manuscript ID	bmjopen-2023-072542.R2
Article Type:	Protocol
Date Submitted by the Author:	12-Jun-2023
Complete List of Authors:	Xu, Pusheng; Sun Yat-Sen University Zhongshan Ophthalmic Center State Key Laboratory of Ophthalmology<br>Ng, Kityee; Sun Yat-Sen University Zhongshan Ophthalmic Center State Key Laboratory of Ophthalmology<br>Jin, Ling; Sun Yat-Sen University Zhongshan Ophthalmic Center State Key Laboratory of Ophthalmology<br>Young, Charlotte; Albany Medical College<br>Liu, Siyuan; Sun Yat-Sen University Zhongshan Ophthalmic Center State Key Laboratory of Ophthalmology<br>Liang, Xiaolin; Sun Yat-Sen University Zhongshan Ophthalmic Center State Key Laboratory of Ophthalmology<br>Liu, Zhenzhen; Sun Yat-Sen University Zhongshan Ophthalmic Center State Key Laboratory of Ophthalmology<br>Zhang, Xinyu; Sun Yat-Sen University Zhongshan Ophthalmic Center State Key Laboratory of Ophthalmology<br>Jin, Guangming; Sun Yat-Sen University Zhongshan Ophthalmic Center State Key Laboratory of Ophthalmology<br>Zheng, Danying; Sun Yat-Sen University Zhongshan Ophthalmic Center State Key Laboratory of Ophthalmology,
**Primary Subject Heading**:	Ophthalmology
Secondary Subject Heading:	Medical management, Ophthalmology, Research methods
Keywords:	Cataract and refractive surgery < OPHTHALMOLOGY, Medical ophthalmology < OPHTHALMOLOGY, Paediatric ophthalmology < PAEDIATRIC SURGERY, Ophthalmology < SURGERY

SCHOLARONE™
Manuscripts

Visual Prognosis and Complications of Congenital Ectopia Lentis: Study Protocol for a Hospital-Based Cohort in Zhongshan Ophthalmic Center

Authors

Pusheng Xu, MD[1†]

Kityee Ng, MD[1†]

Ling Jin, MSc[1]

Charlotte Aimee Young, BS[2]

Siyuan Liu, MD[1]

Xiaolin Liang, MD[1]

Zhenzhen Liu, MD, PhD[1]

Xinyu Zhang, MD, PhD[1]

Guangming Jin, MD, PhD[1*]

Danying Zheng, MD[1*]

Institutions

1. State Key Laboratory of Ophthalmology, Zhongshan Ophthalmic Center, Sun Yat-sen University, Guangdong Provincial Key Laboratory of Ophthalmology and Visual Science, Guangdong Provincial Clinical Research Center for Ocular Diseases, Guangzhou 510060, China.

For peer review only

2. Albany Medical College, 49 New Scotland Ave Albany, New York, Unite States.

† Pusheng Xu and Kityee Ng are joint first authors.

***Corresponding Author:**

Guangming Jin, MD, PhD.

State Key Laboratory of Ophthalmology, Zhongshan Ophthalmic Center, Sun Yat-sen University, Guangdong Provincial Key Laboratory of Ophthalmology and Visual Science, Guangdong Provincial Clinical Research Center for Ocular Diseases, Guangzhou 510060, China.

Email: jingm@mail2.sysu.edu.cn

Professor Danying Zheng, MD.

State Key Laboratory of Ophthalmology, Zhongshan Ophthalmic Center, Sun Yat-sen University, Guangdong Provincial Key Laboratory of Ophthalmology and Visual Science, Guangdong Provincial Clinical Research Center for Ocular Diseases, Guangzhou 510060, China.

Email: zhengdyy@163.com

Financial support

This study was supported by the National Natural Science Foundation of China (81873673, 81900841), and the Basic and Applied Basic Research Foundation of Guangdong Province (2021A1515011673, 2022A1515011181).

For peer review only

Disclosures: None of the authors has a financial or proprietary interest in any material or method mentioned.

Number of Figures: 1

Number of Supplemental Tables: 1

Word count: 2699

For peer review only

Visual Prognosis and Complications of Congenital Ectopia Lentis: Study Protocol for a Hospital-Based Cohort in Zhongshan Ophthalmic Center

ABSTRACT

Introduction Congenital ectopia lentis (CEL) is a rare ocular disease characterized by the dislocation or displacement of the lens. Patients with mild lens dislocations can be treated with conservative methods (e.g., corrective eyeglasses or contact lenses). In contrast, patients with severe CEL usually require surgical management. However, few studies have focused on the visual prognosis and complications in conservative and surgical management patients. This study aims to investigate the prognosis and complications in CEL patients with conservative and surgical management, which is vital for CEL management, especially the choice of surgical timing and surgical method.

Methods and analysis A cohort study will be conducted at Zhongshan Ophthalmic Center. We plan to recruit 604 participants diagnosed with CEL and aged ≥ 3 years old. Patients with mild lens subluxation and stable visual conditions will be included in the non-surgical group and follow-up at 1, 2 and 3 years after enrollment. Patients with severe lens subluxation that accept CEL surgery will be included in the surgical group. Different surgical techniques, including phacoemulsification and in-the-bag intraocular lens implantation (with or without capsular tension ring), transscleral fixation, will be used depending on the severity of dislocation. Patients will be followed up at 3 months, and 1, 2 and 3 years postoperatively. Over a 5-year follow-up period, patients will receive a detailed ocular examination, including optometry, biological measurement, specular microscopy, ultrasound biomicroscopy, anterior segment and posterior segment optical coherence tomography (OCT), OCT angiography (OCTA), echocardiography and questionnaires on vision-related quality of life. The primary outcome is the change of best corrected visual acuity and the incidence of complications in both groups.

Ethics and dissemination Ethics approval was obtained from the ethics committee of the Zhongshan Ophthalmic Center (number: 2022KYPJ207). Study findings will be published in a peer-reviewed journal.

For peer review only

Trial registration number NCT05654025.

For peer review only

Strengths and limitations of this study

- This study is a cohort of congenital ectopia lentis (CEL) in the Chinese population with a relatively large sample size and a comparatively long (5 years) follow-up.
- This study assesses important ophthalmic measurements with significant clinical implications, including visual acuity, intraocular pressure, axial length, high order aberrations, choroidal thickness and choriocapillaris flow deficits.
- Important systemic indicators of Marfan syndrome are measured, including the metacarpophalangeal joint length, incidence of valvular heart disease, aortic root diameter and Z-score.
- In this study, CEL in age less than 3 years old are excluded and participants could be lost to follow-up after surgery.
- This study focuses on individuals with CEL but different subtypes of CEL may not be classified.

For peer review only

INTRODUCTION

Congenital ectopia lentis (CEL) is a rare ocular disease characterized by the dislocation or displacement of the lens. Several systemic diseases have been reported to be associated with CEL, such as Marfan syndrome, homocystinuria, Weill-Marchesani syndrome and Ehlers-Danlos syndrome.[1] However, some patients with CEL may have no known systemic manifestations.[2]

Patients with mild lens dislocations can be treated with conservative methods (e.g., corrective eyeglasses or contact lenses).[3] However, progressive subluxation or complete dislocation of the lens can cause a high degree of myopia or astigmatism, even amblyopia.[4] Surgical intervention is crucial in the management of patients with severe CEL.[5] Currently, there is no unified standard for operation timing for CEL. A previous study recommended surgical intervention when the near vision of children was less than 0.4 in LogMAR acuity.[6] However, some researchers suggested that surgery should be performed when the best corrected visual acuity (BCVA) is less than 0.3 or monocular diplopia occurs.[4 7] Few studies have focused on the refractive change and visual prognosis in CEL patients both with conservative management and surgical management.[8]

Several surgical techniques have been reported over the past decades, such as lensectomy, phacoemulsification without intraocular lens (IOL) implantation, phacoemulsification and IOL implantation (with or without capsular tension ring) and various transscleral fixation of IOL.[9-11] However, the safety and efficacy of these techniques have not been fully validated so far, especially in the Chinese population. Herein we will conduct this cohort study at the Zhongshan Ophthalmic Center, one of the biggest ophthalmic hospitals in China.[12] All children diagnosed with CEL will be followed up for at least three years. Long-term changes in best-corrected visual acuity and the incidence of complications will be evaluated in patients with conservative management and surgical management.

METHODS AND ANALYSIS

For peer review only

This study will be conducted from 5 December 2022 to 5 December 2027. The study was registered on Clinicaltrials.gov (NCT05654025).

Objective

The study is designed as a prospective clinical trial to investigate the prognosis and complications in CEL patients with conservative and surgical management. The aim is to serve as a reference for disease management, specifically for choice of surgical timing and surgical method.

Study design

An approximately 5-year cohort study will be conducted at Zhongshan Ophthalmic Centre, Guangzhou, China. This study will adhere to the Declaration of Helsinki, and ethics have been approved by the ethics committee of the Zhongshan Ophthalmic Center (number: 2022KYPJ207). This study is designed used the SPIRIT reporting guidelines.[13]

Eligibility criteria

Inclusion criteria

1. Diagnosed with congenital lens dislocation and age ≥ 3 years old.

2. Agree to participate in this study with written informed consent from patients or legal guardians.

Exclusion Criteria

1. History of ophthalmic trauma or other ophthalmic surgeries.

2. Combined with other ophthalmic diseases such as primary glaucoma, uveitis and corneal disease.

3. Patients who could not cooperate in the examinations.

For peer review only

Study setting and participants

This cohort study will be conducted at Zhongshan Ophthalmic Center, one of the largest ophthalmic hospitals in China. This study aims to investigate the visual prognosis and complications in CEL patients with different managements. Patients aged three years or above diagnosed with CEL will be recruited from Zhongshan Ophthalmic Center. Moreover, each participant will be followed for at least three years.

Once the participants meet the requirement of our eligibility criteria, they will be asked to join the WeChat (an instant messaging tool) group on the phone and be provided with informed consent. Interested participants or their guardians will sign the consent form and, if appropriate, will complete a thorough ocular examination and systemic evaluations.

Recruitment

The outpatient clinics will carry out the first screening at Zhongshan Ophthalmic Center. Potential participants will be further confirmed by eligibility and recruited at Zhongshan Ophthalmic Center for clinical trials. One of our researchers will contact the participants and explain the trial process in detail to ensure the participants or guardians fully understand the whole study. Once they agree and sign the consent form, further information will be provided, including the purpose of the study, examinations, the importance of follow-up time and duration, and possible risk in treatment. Then the trial will proceed subsequently.

Sample size

Sample size will be estimated as follows: Assuming the incidence of complications in CEL patients is 15%, and the margin of error is 20%. For a 5% significance level, $Z_{\alpha/2}$ is 1.96 for the two-tailed alternative hypothesis. Sample size = $(Z_{\alpha/2})^2 \cdot P(1-P)*1/E^2 = (1.96)^2 \cdot 15\% \cdot (1-15\%)/(15\% \cdot 20\%)=544$. Assuming the loss ratio of 10%, the adjusted sample size will be 544/(1-0.1) = 544/0.9=

604.

Preoperative management

All patients diagnosed with CEL will be followed up since the enrollment. The surgery will be performed when either of the following situations occurs: 1) BCVA < 0.3 in LogMAR acuity; 2) monocular diplopia; 3) progressive subluxation of the lens affecting the pupillary axis; 4) complicated with severe cataract or secondary glaucoma or corneal endothelial decompensation or retinal detachment.[8]

Preoperative medication

All patients will be routinely administered levofloxacin or tobramycin eye drops (four times a day for three days) before surgery to minimize the risk of infection. An intramuscular injection of ethamsylate (1mg/kg)[14] will be used 30 minutes before surgery to reduce bleeding.

Anaesthesia

General anaesthesia or retrobulbar anaesthesia will be used according to the standard clinical routine.

Surgery methods management

The choice of surgical methods depends on the degree of ectopia lentis and the state of zonules. If the extent of the unhealthy zonules (broken or weak) ≤ 180°, phacoemulsification and in-the-bag IOL implantation (with or without capsular tension ring) will be used. Otherwise, the capsular bag will be removed, and IOL will be fixed through transscleral fixation. Surgery will be performed by the same surgeon (Dr. DY Zheng). Rayner 920H/970C or Sensar AR40e will be used as the implanted IOL.

The surgical techniques for patients who received in-the-bag IOL

For peer review only

implantation are as follows: A 3.0 mm corneal tunnel incision will be made at 12 o'clock. Then, a continuous circular capsulorhexis will be performed manually. Iris hooks will stabilize the bag, and the lens will be aspirated with a phacoemulsifer. IOL will be implanted in-the-bag, and the capsular tension ring will be implanted when the IOL cannot be stably fixed.

For patients who received transscleral fixation of IOL, the surgical techniques will be performed as the previous study described.[8] In brief, a 3.0 mm corneal tunnel incision will be made at 12 o'clock. Then, a continuous circular capsulorhexis will be performed manually, and the capsular bag will be removed after the phacoaspiration. Transscleral fixation of IOL will be performed with the two IOL haptic sutured by 8-0 polypropylene at 2.0 mm posterior to the corneal limbus. Anterior vitrectomy will be performed when severe vitreous prolapse occurs.

For peer review only

Postoperative management

After surgery, tobramycin and dexamethasone eye drops (four times a day) and ointment (once every night) will be routinely administered for one week. If intraocular pressure (IOP) is higher than 25 mmHg, topical IOP-lowering medication will be used. If IOP is higher than 40 mmHg, an intravenous drip of 20% mannitol will be used.[15] In case necessary, anterior chamber drainage will be performed through the side incision.

Outcome measures

Primary outcome

The primary outcome is the change of best corrected visual acuity (BCVA) and the incidence of complications (time frame: Non-surgical group is evaluated at the first visit and 1, 2, 3 years after enrollment. Surgical group is evaluated at preoperation, 3 months, and 1, 2, 3 years postoperatively)

Secondary outcomes

1. Change of axial length (time frame: Non-surgical group is evaluated at the

first visit and 1, 2, 3 years after enrollment. Surgical group is evaluated at preoperation, 3 months, and 1, 2, 3 years postoperatively)

2. High order aberrations (time frame: Non-surgical group is evaluated at the first visit and 1, 2, 3 years after enrollment. Surgical group is evaluated at preoperation, 3 months, and 1, 2, 3 years postoperatively)

3. Central cornea endothelial cell loss (time frame: Preoperation, 3 months, and 1, 2, 3 years postoperatively)

4. The state of zonules (time frame: Non-surgical group is assessed at the first visit and 1, 2, 3 years after enrollment. Surgical group will be assessed preoperation)

5. Anterior chamber angle (time frame: Non-surgical group is assessed at the first visit and 1, 2, 3 years after enrollment. Surgical group will be assessed preoperation, 3months, and 1, 2, 3 years postoperatively)

6. Tilt and eccentricity of intraocular lens (time frame: 3 months, and 1, 2, 3 years postoperatively)

7. Intraocular pressure (time frame: Non-surgical group is evaluated at the first visit and 1, 2, 3 years after enrollment. Surgical group is evaluated at preoperation, 3 months, and 1, 2, 3 years postoperatively)

8. Aortic root diameter (time frame: Non-surgical group is evaluated at the first visit and 1, 2, 3 years after enrollment. Surgical group is evaluated at preoperation and 1, 2, 3 years postoperatively)

9. Aortic Root (Sinuses of Valsalva) Z-score, adjusted by body-surface-area (Z-score) (time frame: Non-surgical group is evaluated at the first visit and 1, 2, 3 years after enrollment. Surgical group is evaluated at preoperation and 1, 2, 3 years postoperatively)

10. Incidence of valvular heart disease (time frame: Non-surgical group is evaluated at the first visit and 1, 2, 3 years after enrollment. Surgical group is evaluated at preoperation and 1, 2, 3 years postoperatively)

11. Body mass index (BMI) (time frame: Non-surgical group is evaluated at the first visit and 1, 2, 3 years after enrollment. Surgical group is evaluated at preoperation and 1, 2, 3 years postoperatively)

12. Metacarpophalangeal joint length (time frame: Non-surgical group is evaluated at the first visit and 1, 2, 3 years after enrollment. Surgical group is evaluated at preoperation and 1, 2, 3 years postoperatively.)

13. Choroidal thickness (time frame: Non-surgical group is evaluated at the first visit and 3 years after enrollment. Surgical group is evaluated at preoperation and 3 years postoperatively)

14. Choriocapillaris flow deficits (time frame: Non-surgical group is evaluated

For peer review only

at the first visit and 3 years after enrollment. Surgical group is evaluated at preoperation and 3 years postoperatively)

15. Genetic mutation state of patients (time frame: preoperation)

16. Vision-related quality of life (time frame: Non-surgical group is evaluated at the first visit and 3 years after enrollment. Surgical group is evaluated at preoperation and 3 years postoperatively)

Examinations

Baseline data and follow-up examination items are as follows:

1. Demographic characteristics, including name, gender, and date of birth.
2. Slit-lamp examination: Slit-lamp examination (BQ-900, Haag Streit, Switzerland) and fundus examination will be performed at each visit.
3. Visual acuity and refraction: the uncorrected visual acuity and best corrected visual acuity (BCVA) will be evaluated with ETDRS LogMAR visual acuity chart (Precision Vision, Villa Park, Illinois, USA) at a test distance of 4.0m. The refractive error will be determined by subjective refraction following an objective measurement. Spherical equivalent (SE) will be obtained with the calculation of spherical power plus half of the cylindrical power.
4. Axial length measures: Axial length will be measured using IOLMaster 700 (Carl Zeiss AG, Jena, Germany).
5. High-order aberrations will be assessed using Nidek OPD-Scan III (Gamagori, Japan).
6. Corneal endothelial cell counting and morphology will be detected using an endokeratoscope (SP-2000P, Topcon, Japan).
7. The state of zonules will be assessed clock by clock with ultrasound biomicroscopy (UBM). The clock range of zonular disruption or loosening will be recorded. And this results will be confirmed using slit-lamp photography after dilation.
8. The structure of the anterior chamber angle will be examined using a Tomey Casia 2 anterior segment optical coherence tomography (OCT) (Tomey, Tokyo, Japan).[16]
9. The tilt and eccentricity of IOL will be evaluated using Pentacam AXL (Oculus, Germany).[17]

For peer review only

10. Intraocular pressure (IOP) measures: The IOP in the patient's eyes will be measured using a non-contact-tonometer at each visit, and the average of the three measures will be taken.

11. Echocardiography examination: This examination will be performed using doppler echocardiography (HP/Philips Sonos 5500, Philips, Bothell, WA, USA). A skilled technician will measure the aortic root diameter. Z-Score will be calculated using the Marfan foundation's calculator (https://marfan.org/dx/zscore-children/). Normal Z-score ranges from -2 to 2. A dilated aortic root is defined as a Z-score ≥ 2.0. A larger Z-score is associated with an increased risk of aortic complications such as dissection, rupture, and valvular regurgitation. An experienced cardiologist will determine the presence or absence of heart valve disease (HVD).

12. Body mass index (BMI). BMI is a person's weight in kilograms divided by the square of height in meters. The normal range for the Chinese population is 18.5 to 23.9. A higher BMI can indicate higher levels of body fat.

13. Metacarpophalangeal joint length will be measured in hand radiograph.

14. The choroidal thickness and choriocapillaris flow deficits will be measured using posterior segment optical coherence tomography (OCT) and optical coherence tomography angiography (OCTA) (Zeiss Cirrus 5000 with AngioPlex).

15. Gene detection: Genomic DNA from each subject in the study will be analyzed by whole-exome sequencing (WES) to detect mutations and diagnosis.

16. Vision-related quality of life will be assessed using the Pediatric Eye Questionnaire (PedEyeQ).[18]

17. Adverse events or complications will be collected at each visit.

Workflow

The workflow will be carried out in the order mentioned above, starting with patients recruitment and ending with a minimum of 3 years follow-up. (Figure 1). The non-surgical group will be followed up 1, 2, and 3 years after enrollment. The surgical group will be followed up at 3 months, and 1, 2, 3 years postoperatively. The examinations planned for each follow-up time point are

For peer review only

shown in Supplemental table 1.

Data collection and management

The case report form (CRF) will collect basic demographic and clinical information. Results of eye examinations will be recorded on paper, and questionnaires will be filled out in the paper form. Original data will enter the lens dislocation case database at the Zhongshan Ophthalmic Center. After the study, relevant documents will be stored securely at the Zhongshan Ophthalmic Center for ten years for specific scientific research purposes.

Statistical analysis plan

Statistical analysis will be performed using Stata 15.0 (StataCorp, College Station, Texas, USA). Quantitative data conforming to a normal distribution will be described as the mean ± SD. The difference between the baseline and follow-up data will be compared by one-way analysis of variance (ANOVA). The median ± interquartile range will describe the quantitative data of the skewness distribution. The Wilcoxon signed-rank test will be used to compare these data's differences. For qualitative data described as proportions, the chi-square test or Fisher's exact test will be used to compare the differences between groups. $P<0.05$ is considered statistically significant. The differences and 95% confidence interval (CI) in the changes of BCVA and other parameters will be calculated. The univariable linear regression model will estimate these changes and their associated factors. All variables with $P<0.05$ in the univariable regression analysis will be included in the multivariable linear regression model.

Study monitoring

Clinical examiners will regularly check each patient's informed consent and eligibility to ensure that all CRFs are correct and in accordance with the original data. All errors and omissions should be recorded and corrected. The examiner should ensure every participant's withdrawal and loss of follow-up are recorded and explained in CRF, and all adverse events are recorded. The surgical operations in this project are standard methods and do not pose significant risks.

For peer review only

The principal risks include general anaesthesia adverse effects and adverse drug reactions. This cohort study will be conducted under the guidance of the ethics committee of the Zhongshan Ophthalmic Center.

Patient and public involvement

Neither the patients nor the public is involved in our research's design, conduct, reporting, or dissemination plans.

Ethics and dissemination

Ethics approval was obtained from the ethics committee of the Zhongshan Ophthalmic Center (number: 2022KYPJ207). Signed consents will be obtained from the legal guardians of participants after they have been informed of the study workflow and their right to withdraw from the cohort study. This project has been designed following the principles of the Declaration of Helsinki.

The content of this cohort study is confidential information. Clinical records and data sets will be kept at the Zhongshan Ophthalmic Center in strict confidence and will only be assessed by the study investigators and authorized personnel. The results without personal data will be disseminated through peer-reviewed publications and conference presentations.

For peer review only

Contributors PX, KN, LJ, ZL, XZ, GJ, and DZ conceived and designed the study. PX, KN, SL, and XL wrote the draft. CY, GJ and DZ revised the draft. GJ will lead the statistical analysis. ZL, XZ, GJ and DZ will oversee data acquisition and implementation on-site. All authors reviewed and approved the final manuscript.

Funding This study was supported by the National Natural Science Foundation of China (81873673, 81900841) and the Basic and Applied Basic Research Foundation of Guangdong Province (2021A1515011673, 2022A1515011181).

Competing interests None of the authors has a financial or proprietary interest in any material or method mentioned.

Data Availability Statement Data are available upon reasonable request

Patient consent for publication Not applicable.

Provenance and peer review Not commissioned; externally peer reviewed.

For peer review only

Reference

1. Sadiq MA, Vanderveen D. Genetics of ectopia lentis. *Semin Ophthalmol* 2013;28(5-6):313-20. doi: 10.3109/08820538.2013.825276
2. Guo D, Yang F, Zhou Y, et al. Novel ADAMTSL4 gene mutations in Chinese patients with isolated ectopia lentis. *Br J Ophthalmol* 2022 doi: 10.1136/bjophthalmol-2021-320475
3. Esfandiari H, Ansari S, Mohammad-Rabei H, et al. Management Strategies of Ocular Abnormalities in Patients with Marfan Syndrome: Current Perspective. *J Ophthalmic Vis Res* 2019;14(1):71-77. doi: 10.4103/jovr.jovr_29_18
4. Rezar-Dreindl S, Stifter E, Neumayer T, et al. Visual outcome and surgical results in children with Marfan syndrome. *Clin Exp Ophthalmol* 2019;47(9):1138-45. doi: 10.1111/ceo.13596
5. Chee S-P, Ti S-E, Chan NS-W. Management of the subluxated crystalline lens: A review. *Clin Exp Ophthalmol* 2021;49(9):1091-101. doi: 10.1111/ceo.13975
6. Matsuo T. How far is observation allowed in patients with ectopia lentis? *Springerplus* 2015;4:461. doi: 10.1186/s40064-015-1239-5
7. Català-Mora J, Cuadras D, Díaz-Cascajosa J, et al. Anterior iris-claw intraocular lens implantation for the management of nontraumatic ectopia lentis: long-term outcomes in a paediatric cohort. *Acta Ophthalmol* 2017;95(2):170-74. doi: 10.1111/aos.13192
8. He J, Lian Z, Cao Q, et al. Longitudinal Changes of Axial Length and Associated Factors in Congenital Ectopia Lentis Patients. *J Ophthalmol* 2022;2022:4032283. doi: 10.1155/2022/4032283
9. Hoffman RS, Snyder ME, Devgan U, et al. Management of the subluxated crystalline lens. *J Cataract Refract Surg* 2013;39(12):1904-15. doi: 10.1016/j.jcrs.2013.09.005

10. Hsu HY, Edelstein SL, Lind JT. Surgical management of non-traumatic pediatric ectopia lentis: A case series and review of the literature. *Saudi J Ophthalmol* 2012;26(3):315-21. doi: 10.1016/j.sjopt.2012.05.001
11. Stephenson KA, O'Keefe M, Keegan DJ. Surgical management of non-syndromic ectopia lentis. *International Journal of Ophthalmology* 2020;13(7):1156-60. doi: 10.18240/ijo.2020.07.21
12. Chan C-C, Liu MM, Tsai JC. The first Western-style hospital in China. *Arch Ophthalmol* 2011;129(6):791-97. doi: 10.1001/archophthalmol.2011.120
13. Chan A-W, Tetzlaff JM, Gøtzsche PC, et al. SPIRIT 2013 explanation and elaboration: guidance for protocols of clinical trials. *BMJ* 2013;346:e7586. doi: 10.1136/bmj.e7586
14. Chinese PC. Pharmacopoeia of the People's Republic of China. *China Medical Science Press: Beijing, China* 2020
15. Ramachandra V, Chandran P, Philip R, et al. Effect of Mannitol on Intraocular Pressure in Vitrectomized and Nonvitrectomized Eyes: A Prospective Comparative Study. *J Glaucoma* 2019;28(4):318-20. doi: 10.1097/IJG.0000000000001158
16. Qi H, Jin G, Zou M, et al. Characteristics of Anterior Segment in Congenital Ectopia Lentis: An SS-OCT Study. *J Ophthalmol* 2022;2022:6128832. doi: 10.1155/2022/6128832
17. Ye H, Liu Z, Cao Q, et al. Evaluation of Intraocular Lens Tilt and Decentration in Congenital Ectopia Lentis by the Pentacam Scheimpflug System. *J Ophthalmol* 2022;2022:7246730. doi: 10.1155/2022/7246730
18. Leske DA, Hatt SR, Castañeda YS, et al. Validation of the Pediatric Eye Questionnaire in Children with Visual Impairment. *Am J Ophthalmol* 2019;208:124-32. doi: 10.1016/j.ajo.2019.07.018

For peer review only

Figure legends:

Figure 1. Flowchart of the study. CEL, congenital ectopia lentis. * The surgery will be performed when either of the following situations occurs.

For peer review only

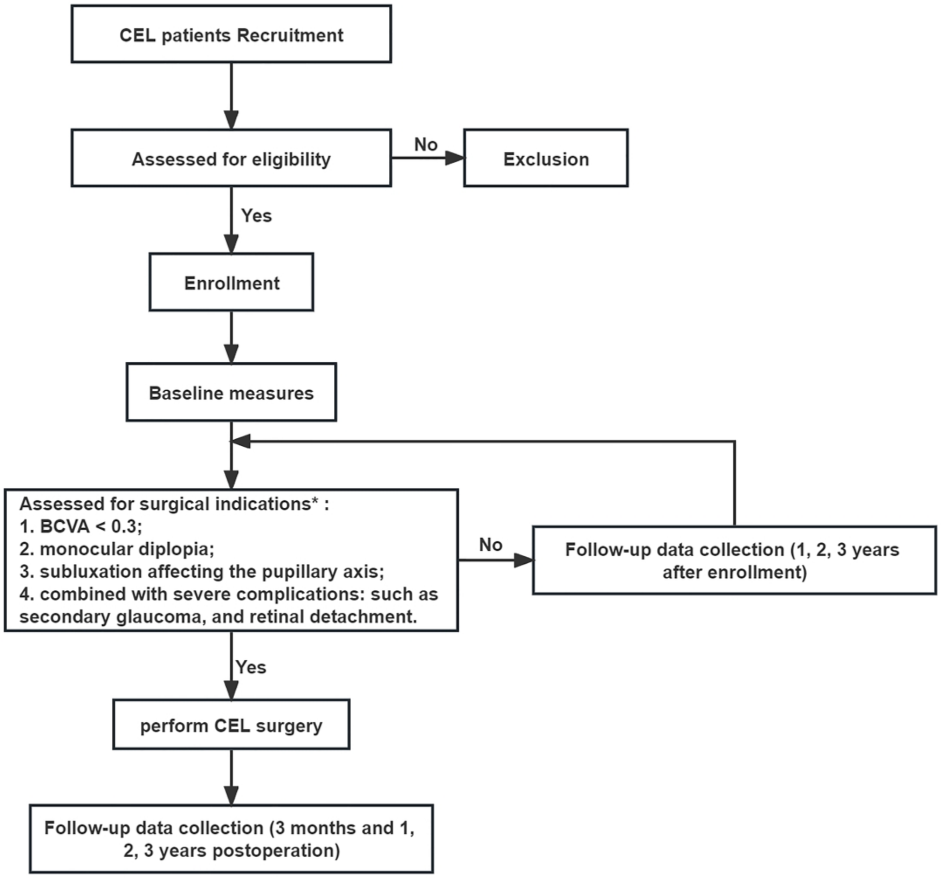

Figure 1. Flowchart of the study. CEL, congenital ectopia lentis. * The surgery will be performed when either of the following situations occurs.

500x469mm (300 x 300 DPI)

Supplemental table 1. Complete overview of follow-up and examinations.

Examination	*Time points of non-surgical group*				*Time points of surgical group*				
	Baseline	*1 y*	*2 y*	*3 y*	*Preoperation**	*3 m*	*1 y*	*2 y*	*3 y*
Demographic data	✓				✓				
Slit-lamp examination	✓	✓	✓	✓	✓	✓	✓	✓	✓
Visual acuity and refraction	✓	✓	✓	✓	✓	✓	✓	✓	✓
Axial length	✓	✓	✓	✓	✓	✓	✓	✓	✓
High-order aberrations	✓	✓	✓	✓	✓	✓	✓	✓	✓
Specular microscopy					✓	✓	✓	✓	✓
Anterior segment OCT	✓	✓	✓	✓	✓	✓	✓	✓	✓
UBM	✓	✓	✓	✓	✓				
Slit-lamp photography	✓	✓	✓	✓	✓				
Pentacam	✓	✓	✓	✓	✓	✓	✓	✓	✓
IOP	✓	✓	✓	✓	✓	✓	✓	✓	✓
Echocardiography	✓	✓	✓	✓	✓		✓	✓	✓
BMI	✓	✓	✓	✓	✓		✓	✓	✓
Hand radiograph	✓	✓	✓	✓	✓		✓	✓	✓
Posterior segment OCT	✓			✓	✓				✓
Gene detection†	✓				✓				
PedEyeQ	✓			✓	✓				✓
Adverse events	✓	✓	✓	✓	✓	✓	✓	✓	✓

** Preoperation examninations will not be repeated if the patient has just completed follow-up and meets the surgical indications.*

† Gene detection will not be repeated if the patient already has a valid genetic testing report.

OCT, optical coherence tomography; UBM, ultrasound biomicroscopy; IOP, intraocular pressure; BMI, body mass index; PedEyeQ, the Pediatric Eye Questionnaire; y, years; m, months.

For peer review only

Reporting checklist for protocol of a clinical trial.

Based on the SPIRIT guidelines.

Instructions to authors

Complete this checklist by entering the page numbers from your manuscript where readers will find each of the items listed below.

Your article may not currently address all the items on the checklist. Please modify your text to include the missing information. If you are certain that an item does not apply, please write "n/a" and provide a short explanation.

Upload your completed checklist as an extra file when you submit to a journal.

In your methods section, say that you used the SPIRIT reporting guidelines, and cite them as:

Chan A-W, Tetzlaff JM, Gøtzsche PC, Altman DG, Mann H, Berlin J, Dickersin K, Hróbjartsson A, Schulz KF, Parulekar WR, Krleža-Jerić K, Laupacis A, Moher D. SPIRIT 2013 Explanation and Elaboration: Guidance for protocols of clinical trials. BMJ. 2013;346:e7586

For peer review only

		Reporting Item	Page Number
**Administrative information**			
Title	#1	Descriptive title identifying the study design, population, interventions, and, if applicable, trial acronym	1
Trial registration	#2a	Trial identifier and registry name. If not yet	3,5,8-9,18

		registered, name of intended registry	
Trial registration: data set	#2b	All items from the World Health Organization Trial Registration Data Set	9
Protocol version	#3	Date and version identifier	n/a, 2022-Dec-27 Original
Funding	#4	Sources and types of financial, material, and other support	18
Roles and responsibilities: contributorship	#5a	Names, affiliations, and roles of protocol contributors	18
Roles and responsibilities: sponsor contact information	#5b	Name and contact information for the trial sponsor	18
Roles and responsibilities: sponsor and funder	#5c	Role of study sponsor and funders, if any, in study design; collection, management, analysis, and interpretation of data; writing of the report; and the decision to submit the report for publication, including whether they will have ultimate authority over any of these activities	n/a, study sponsor and funders don't participate in the study
Roles and responsibilities: committees	#5d	Composition, roles, and responsibilities of the coordinating centre, steering committee, endpoint adjudication committee, data management team,	16

For peer review only

		and other individuals or groups overseeing the trial, if applicable (see Item 21a for data monitoring committee)	
**Introduction**			
Background and rationale	#6a	Description of research question and justification for undertaking the trial, including summary of relevant studies (published and unpublished) examining benefits and harms for each intervention	7
Background and rationale: choice of comparators	#6b	Explanation for choice of comparators	7
Objectives	#7	Specific objectives or hypotheses	7
Trial design	#8	Description of trial design including type of trial (eg, parallel group, crossover, factorial, single group), allocation ratio, and framework (eg, superiority, equivalence, non-inferiority, exploratory)	7
**Methods: Participants, interventions, and outcomes**			
Study setting	#9	Description of study settings (eg, community	9

For peer review only

		clinic, academic hospital) and list of countries where data will be collected. Reference to where list of study sites can be obtained	
Eligibility criteria	#10	Inclusion and exclusion criteria for participants. If applicable, eligibility criteria for study centres and individuals who will perform the interventions (eg, surgeons, psychotherapists)	8-9
Interventions: description	#11a	Interventions for each group with sufficient detail to allow replication, including how and when they will be administered	10-11
Interventions: modifications	#11b	Criteria for discontinuing or modifying allocated interventions for a given trial participant (eg, drug dose change in response to harms, participant request, or improving / worsening disease)	10
Interventions: adherance	#11c	Strategies to improve adherence to intervention protocols, and any procedures for monitoring adherence (eg, drug tablet return; laboratory tests)	9
Interventions: concomitant care	#11d	Relevant concomitant care and interventions that are permitted or prohibited during the trial	9
Outcomes	#12	Primary, secondary, and other outcomes, including the specific measurement variable (eg, systolic blood pressure), analysis metric (eg, change from baseline, final value, time to event),	11-13

For peer review only

		method of aggregation (eg, median, proportion), and time point for each outcome. Explanation of the clinical relevance of chosen efficacy and harm outcomes is strongly recommended	
Participant timeline	#13	Time schedule of enrolment, interventions (including any run-ins and washouts), assessments, and visits for participants. A schematic diagram is highly recommended (see Figure)	8
Sample size	#14	Estimated number of participants needed to achieve study objectives and how it was determined, including clinical and statistical assumptions supporting any sample size calculations	9-10
Recruitment	#15	Strategies for achieving adequate participant enrolment to reach target sample size	9
**Methods: Assignment of interventions (for controlled trials)**			
Allocation: sequence generation	#16a	Method of generating the allocation sequence (eg, computer-generated random numbers), and list of any factors for stratification. To reduce predictability of a random sequence, details of any	n/a, non-random cohort

For peer review only

		planned restriction (eg, blocking) should be provided in a separate document that is unavailable to those who enrol participants or assign interventions	
Allocation concealment mechanism	#16b	Mechanism of implementing the allocation sequence (eg, central telephone; sequentially numbered, opaque, sealed envelopes), describing any steps to conceal the sequence until interventions are assigned	n/a, non-random cohort
Allocation: implementation	#16c	Who will generate the allocation sequence, who will enrol participants, and who will assign participants to interventions	n/a, non-random cohort
Blinding (masking)	#17a	Who will be blinded after assignment to interventions (eg, trial participants, care providers, outcome assessors, data analysts), and how	n/a, un-blinded study
Blinding (masking): emergency unblinding	#17b	If blinded, circumstances under which unblinding is permissible, and procedure for revealing a participant's allocated intervention during the trial	n/a, un-blinded study
**Methods: Data collection, management, and analysis**			
Data collection plan	#18a	Plans for assessment and collection of outcome, baseline, and other trial data, including any	15

For peer review only

		related processes to promote data quality (eg, duplicate measurements, training of assessors) and a description of study instruments (eg, questionnaires, laboratory tests) along with their reliability and validity, if known. Reference to where data collection forms can be found, if not in the protocol	
Data collection plan: retention	#18b	Plans to promote participant retention and complete follow-up, including list of any outcome data to be collected for participants who discontinue or deviate from intervention protocols	15
Data management	#19	Plans for data entry, coding, security, and storage, including any related processes to promote data quality (eg, double data entry; range checks for data values). Reference to where details of data management procedures can be found, if not in the protocol	15
Statistics: outcomes	#20a	Statistical methods for analysing primary and secondary outcomes. Reference to where other details of the statistical analysis plan can be found, if not in the protocol	15
Statistics: additional analyses	#20b	Methods for any additional analyses (eg, subgroup and adjusted analyses)	15
Statistics: analysis	#20c	Definition of analysis population relating to	15

For peer review only

population and missing data		protocol non-adherence (eg, as randomised analysis), and any statistical methods to handle missing data (eg, multiple imputation)	
**Methods: Monitoring**			
Data monitoring: formal committee	#21a	Composition of data monitoring committee (DMC); summary of its role and reporting structure; statement of whether it is independent from the sponsor and competing interests; and reference to where further details about its charter can be found, if not in the protocol. Alternatively, an explanation of why a DMC is not needed	16
Data monitoring: interim analysis	#21b	Description of any interim analyses and stopping guidelines, including who will have access to these interim results and make the final decision to terminate the trial	16
Harms	#22	Plans for collecting, assessing, reporting, and managing solicited and spontaneously reported adverse events and other unintended effects of trial interventions or trial conduct	16
Auditing	#23	Frequency and procedures for auditing trial conduct, if any, and whether the process will be independent from investigators and the sponsor	16
**Ethics and dissemination**			

For peer review only

Research ethics approval	#24	Plans for seeking research ethics committee / institutional review board (REC / IRB) approval	16
Protocol amendments	#25	Plans for communicating important protocol modifications (eg, changes to eligibility criteria, outcomes, analyses) to relevant parties (eg, investigators, REC / IRBs, trial participants, trial registries, journals, regulators)	n/a, contact with the corresponding author
Consent or assent	#26a	Who will obtain informed consent or assent from potential trial participants or authorised surrogates, and how (see Item 32)	16
Consent or assent: ancillary studies	#26b	Additional consent provisions for collection and use of participant data and biological specimens in ancillary studies, if applicable	n/a
Confidentiality	#27	How personal information about potential and enrolled participants will be collected, shared, and maintained in order to protect confidentiality before, during, and after the trial	15
Declaration of interests	#28	Financial and other competing interests for principal investigators for the overall trial and each study site	18
Data access	#29	Statement of who will have access to the final trial dataset, and disclosure of contractual agreements that limit such access for investigators	15

For peer review only

Ancillary and post trial care	#30	Provisions, if any, for ancillary and post-trial care, and for compensation to those who suffer harm from trial participation	n/a
Dissemination policy: trial results	#31a	Plans for investigators and sponsor to communicate trial results to participants, healthcare professionals, the public, and other relevant groups (eg, via publication, reporting in results databases, or other data sharing arrangements), including any publication restrictions	18
Dissemination policy: authorship	#31b	Authorship eligibility guidelines and any intended use of professional writers	n/a
Dissemination policy: reproducible research	#31c	Plans, if any, for granting public access to the full protocol, participant-level dataset, and statistical code	n/a

Appendices

Informed consent materials	#32	Model consent form and other related documentation given to participants and authorised surrogates	18
Biological specimens	#33	Plans for collection, laboratory evaluation, and storage of biological specimens for genetic or molecular analysis in the current trial and for future use in ancillary studies, if applicable	n/a

For peer review only

None The SPIRIT Explanation and Elaboration paper is distributed under the terms of the Creative Commons Attribution License CC-BY-NC. This checklist can be completed online using https://www.goodreports.org/, a tool made by the EQUATOR Network in collaboration with Penelope.ai

For peer review only
